# Framework for systems of pediatric well-care visits: a scoping review

**DOI:** 10.1186/s44263-025-00211-4

**Published:** 2025-11-13

**Authors:** Alma Nordenstam, Daniel Helldén, Anna-Theresia Ekman, Margareta Blennow, Grace Ndeezi, Olivia Biermann, Tobias Alfvén

**Affiliations:** 1 Department of Global Public Health, Karolinska Institutet, Stockholm, Sweden; 2 Centre of Excellence for Sustainable Health, Makerere University, Kampala, Uganda and Karolinska Institutet, Stockholm, Sweden; 3 Sachs’ Children and Youth Hospital, Stockholm, Sweden; 4 Department of Pediatrics and Child Health, College of Health Sciences, Makerere University, Kampala, Uganda; 5 Institutes for Transdisciplinary Scholarships, University of Calgary, Calgary, Canada

**Keywords:** Well-care visits, Child health care, Prevention, Integrated care, Child health days, Child health clinics, Social determinants of health

## Abstract

**Background:**

Despite decades of global efforts to reduce under five mortality, 5 million children die before their fifth birthday. Numerous landmark reports have called for integrated approaches to further accelerate reductions, yet interventions are often delivered in silos and, to our knowledge, no global synthesis of the evidence for integrated preventive systems exists. We conducted a scoping review to map the existing literature on systems of well-care visits for children under five years from 2000 to 2023 globally.

**Method:**

A systematic search of relevant databases (MEDLINE, the Cochrane Library, Web of Science, and OVID Global Health) was conducted. In total, 8,945 unique documents were identified and after screening titles and abstracts, a total of 1587 articles were assessed for eligibility through full-text review. The information was extracted based on the WHO’s established health system framework, and from these pillars, a conceptual framework was derived on the components and the potential impact on child health care.

**Results:**

We found 322 eligible articles (217 primary articles, 105 reviews). Among the primary articles, close to 50% focused on high-income countries, while less than a fifth focused on low-income countries. Across regions, systems of preventive well-care visits are organized differently; from health days to home-visits, programs, and clinics. Depending on the context, the content differs (e.g., vaccinations, screenings, parent education) and the contextual challenges and solutions for implementation (e.g., mHealth, scale up existing structures).

**Conclusions:**

This review identifies core components of well-care visit systems, including interventions, workforce composition, and governance structures, alongside key implementation strategies such as the scaling up of existing service delivery models. We introduce a framework for systems of well-care visits. These systems may offer child health care improvements, including enhanced attendance rates, increased service utilization, and decreased health care expenditure, thereby contributing to improved child health care for all.

**Supplementary Information:**

The online version contains supplementary material available at 10.1186/s44263-025-00211-4.

## Background

Worldwide, 85% of all children who die are under the age of 5 [1]. Despite global targets to reduce child mortality through both the Millennium Development Goals (MDGs) followed by the Sustainable Development Goals (SDGs), 5 million children under the age of 5 die each year, mostly from avoidable deaths [2]. Predictions indicate that without accelerated efforts, 54 countries, predominantly from low- and middle-income countries (LMICs), will not reach their under-5 mortality goals by 2030 [2]. Moreover, children’s developmental potential is compromised by diseases, malnutrition and multifactorial poverty, especially during the first years of life, with lasting consequences for their health and wellbeing throughout life [3].

Health systems, often resource restricted, face the challenge of planning and implementing preventive health services for children that utilize resources in the best possible way [4]. Numerous landmark reports have suggested the benefits of integrated approaches [5–7] to preventive child health [5–7]. Integrated approaches have been promoted through the Integrated Management of Childhood Illnesses (IMCI) program developed to integrate preventive care concurrently with curative care [8]. However, whilst IMCI can reduce childhood deaths, the evidence shows limited, if any, improvement on key preventive measures, such as vaccination rates and reductions in wasting and stunting [8].

An increasing number of countries are introducing variants of systems of well-care visits [9]. While lacking a specific definition, these programs include preventive child health interventions such as vaccinations, screenings (e.g., hearing), and supplements (e.g., vitamin A supplement, mosquito nets), but they often also encompass health education and promotion activities, developmental screenings, and addressing concerns for individual children [9].

Recently, the World Health Organization (WHO) and the United Nations International Emergency Fund (UNICEF) published guidance on well-care visits to improve preventive child health care utilizing a life-course approach [9]. Under the umbrella term well-care visits, everything from integrated delivery of preventive care at child days, specific home programs, to clinics are recommended throughout the life-course of the child [9]. Based on expert consultations, the guidance provides a health system integrated preventive child health care, including information such as the types of interventions, health care professionals, and structures of care. Nonetheless, while the effectiveness of many of these interventions to improve child health is well-documented, no global review of the literature for pediatric well-care visits exists.

Few attempts over the past decades have been made to synthesize the evidence on systems of well-care visits. Previous reviews of high-income countries (HICs) [10–15] suggest that preventive child health care models need to be contextualized to the site-specific needs. Only one review has focused on LMICs [16]. The review by Ahmed and Won [16] has a scope of five countries and is based on a qualitative analysis of one country with Demographic Health Survey’s data of the other countries. No global review with both LMICs and HICs has, to our knowledge, been conducted. Thus, there is a paucity of data on how preventive child health care services are constructed and implemented, and the barriers and solutions to successfully do so, in different health care systems and income settings.

We aimed to compile the global evidence on well-care visits for children under 5 to provide a synthesis of studies, characterizing the elements on integrated systems, barriers and solutions for implementation. The evidence provides guidance for researchers, practitioners, and policy-makers in the planning and implementation of well-care visits, thus accelerating the work to implement the guidance from WHO and UNICEF in a global effort to end preventable child mortality and morbidity.

## Methods

We selected a scoping review methodology due to its relative flexibility to incorporate different types of information and to synthesize a broad area of inquiry [17]. The Prisma-ScR checklist [18] was used for reporting (Supplementary material 1: PRISMA-ScR checklist), and due to time constrains no study protocol was published.

### Inclusion and exclusion criteria

Articles were included if their content focused on children under the age of 5. We screened for preventive care. WHO calls for integrated well-care visits. However, no established definition of the minimum number of interventions for this system-approach exists. As such we defined systems of well-care visits as all with multifaceted interventions (i.e., two or more interventions delivered in tandem). Articles were excluded based on irrelevant content, unavailability of full-texts in English, papers other than reviews or original papers, lack of systems (i.e., single interventions), lack of preventive care, or incorrect age range (i.e., children 5 years of age or older). Curative care which is not part of the guidelines of well-care visits and so we also excluded documents who combined curative and preventive care [9]. All articles in English published after the MDGs were introduced, from first of January 2000 up to the date of the literature search were considered for review. To address the lack of a global review of well-care visits across resource settings and regions, we employed no restriction on location. The original search was conducted on 14 June 2021 with an update on 16 June 2023.

### Search strategy

We developed search strategies for MEDLINE, the Cochrane Library, Web of Science, and OVID Global Health to include preventive child health care activities: “child health services” and “children under 5” in MeSH terms or equivalent index for the respective databases, as well as in free text. When databases provided MeSH terms, an additional block of excluding “hospitalization” was added to strengthen focus on primary and preventive care. Separate components of preventive child health care, including immunization programs and screening, were not searched for directly to avoid capturing single interventions. The detailed search strategies for each database are available in Supplementary material 1: Detailed search strategy.

### Literature screening and data extraction

AN screened all the retrieved documents’ titles and abstracts for relevance according to the study aim. This was followed by a full-text review of all relevant articles according to our inclusion criteria. Due to limited resources, approximately 10% of the relevant documents were then randomly selected and double-screened by OB and DH and any discrepancies were resolved through discussion to reach consensus. All documents were divided into primary articles or reviews; a data extraction table was created (Supplementary material 2: Data extraction sheet) for each respective document type. AN extracted general information, contextual information of the study setting, and the population from all included documents (primary articles and reviews). Each review was treated as one document, and due to the heterogeneity of these data, a table of the study settings (e.g., income level, geographical location) was only generated for primary articles.

The activities included in preventive child health care were extracted using the WHO’s established health system framework [19] focusing on health service delivery, health workforce, health information systems, access to essential medicines, health system financing and leadership, and governance. We also assessed for barriers and solutions to the implementation of systems of well-care visits to address the determinants of their implementation (e.g., behavioral, structural and contextual) of well-care visits. Reviews were assessed accordingly, however given the scope of the material the reference lists for the reviews were not assessed. The content of each review was extracted as one document, and when individual studies within the review were described they were not extracted as separate documents. Following this, open coding was applied to identify cross-reference themes across the material which was used in developing the final framework.

All papers were classified according to income status based on the classification of the World Bank in the year of publication [20]. For the primary articles in which multiple countries were present, the median of the countries’ income level was used for classification. Papers with multiple countries present (*n* = 2) were excluded from income status classification. Countries not included in WHO regions (*n* = 2) as well as papers with multiple regions present were excluded (*n* = 2) from the geographical classification.

## Results

**Fig. 1:**
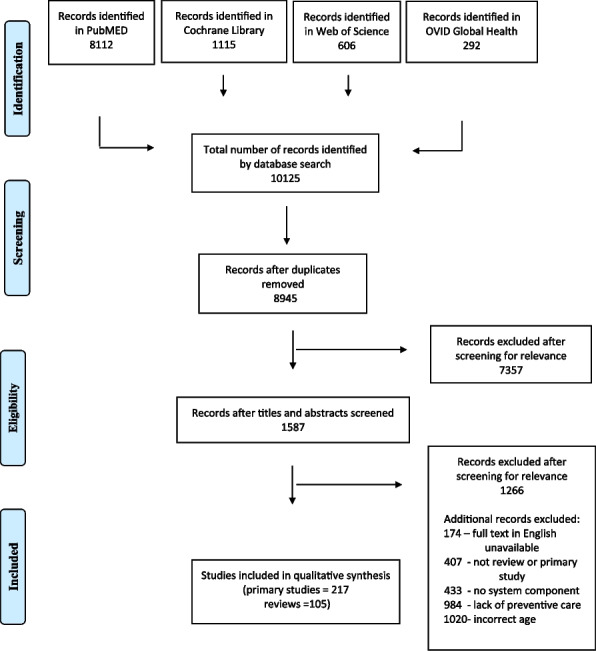
PRISMA flow diagram

We identified a total of 10, 125 documents. After removal of duplicates, 8945 unique documents remained (Fig. 1). Of these, we excluded 7357 after screening titles and abstracts resulting in 1587 articles eligible for full-text review. After applying the inclusion and exclusion criteria, 322 articles were included in the review (217 primary articles and 105 reviews).

### Characteristics of the included articles

Among the primary articles, almost half of them focused on high-income countries, while less than a fifth of the articles focused on low-income countries (Table 1). Over 80% of the articles were either set in an urban environment or in a mix of urban and rural environment. The majority of the studies (54%) used quantitative methods, followed by mixed methods (30%) and qualitative methods (16%) associated with preventive integrated child health care.

**Table 1:** Characteristics of the primary articles included in the scoping review (*n* = 217)

	Primary articles
Income status	*n* (%)
High-income	100 (46)
Upper middle-income	30 (14)
Lower middle-income	46 (21)
Low-income	38 (18)
Study setting	
Urban	76 (34)
Rural	38 (17)
Both	97 (46)
Study method type	
Quantitative	115 (54)
Qualitative	33 (16)
Mixed method	68 (30)
Geographical area	
African Region	72 (34)
Region of the Americas	71 (34)
South-East Asian Region	16 (8)
European Region	25 (12)
Eastern Mediterranean Region	4 (2)
Western Pacific Region	23 (11)

**Fig. 2:**
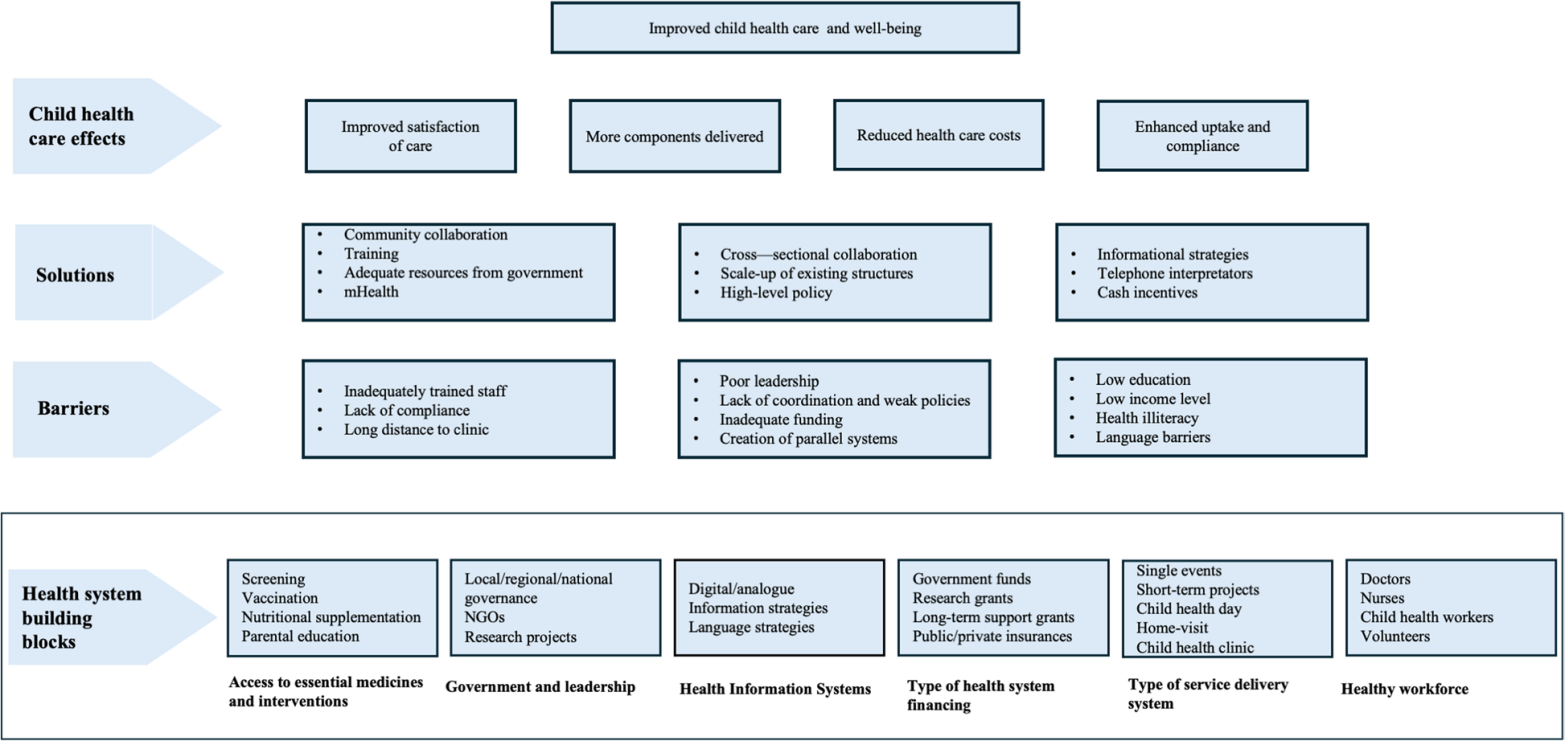
Synthesis of preventive child health systems sorted through the health system building blocks according to the World Health Organization

Further, the literature describes key barriers and solutions for integrated preventive child health care and points to positive child health effects, such as improved satisfaction of care for both parents and caregivers, reduced individual costs, more components delivered within available resources and enhanced uptake. An overview of key concepts regarding integrated preventive child health care in this review is illustrated in Fig. 2.

### Health system building blocks for systems of well-care visits

#### Types of service delivery system

Across regions, well-care visits are organized in various types of health service delivery (Table 2); from health days [21, 22], to home-visits [23–27], and clinics [16, 28–30]. Child health days often operate as campaigns where a local health station is set up to mass-immunize people over a short-time period, lasting from 2 to 3 days to a week [20]. Child health days, a model reported primarily in limited resource settings, has been used to deliver various interventions concurrently [21, 22]. Another structure is home-visits in which a health professional visits the family [23–25, 27, 31]. The number of visits varies, from one right after birth to a continuous program encompassing the first month [32], year [31] or several years [23]. Such visits may be part of the clinics [24] or be a stand-alone structure [23, 32]. Clinics are heterogenous across communities, countries, and regions [29, 30, 33, 34]. In other areas, preventive child health care is located outside of a family’s home at clinics, either as part of curative care [33] or as separate entities that only focuses on preventive care [30, 35]. The clinics provide well-care visits for the children either on a one-to-one basis, or together with group counselling [30], and community events [30]. Similar to health visits, the age-span for the clinics varies and some cover children for the first year [36], others for several years [29, 30] or all years up until the fifth birthday [33, 34].

**Table 2:** Examples of systems for pediatric well-care visits

Child health days Child health days are point-interventions to support the existing health care systems with various components. Traditionally they entail immunization. However, adding additional components whilst immunizing can be a method to provide integrated care under limited resource settings. Usually, child health days occur every 6 months where normal health care workers go out to a rural area for a short period of time (days) to enhance the uptake For example, in Uganda child health days were set up to target children 6–59 months of age for measles immunization, vitamin A supplement, deworming tablets, and insecticide treated bed nets. Temporary outreach posts, and mobile units were used together with existing health facilities to locate the service provision. This resulted in increased uptake in general with use of vitamin A supplements increasing by 30% compared to before when it was not part of child health days [20]
Home visiting programs Home visiting programs involve a health care personnel coming to the family’s house to provide services. The background of the provider varies, from doctors, nurses to child health workers. Programs (local or government-led) may involve a singular visit or be a series of visits during the first week, month, or year(s) of a child’s life. They can be part of a health care clinic’s larger program or separate interventions In Guatemala, the Niños Sanos (“healthy children”) program was set up to deliver newborn home-visits, community education sessions and mother–child interactive groups to promote the development of the child’s health and well-being. The services were provided by childhood nurses or workers that completed a training course pre-program intervention by pediatricians [24]
Child health clinics (programs or permanent) Child health clinics involve a physical place, either mobile or permanent, where children and parents come to visit a health care personnel at a regular basis. The number of visits, and whether they also involve phone calls and home-visits, varies. In some settings, doctors are the key point of contact, whereas in other places it is nurse-led or a multidisciplinary team. Here, children may receive health-checkups, screenings, immunizations, and supplementation of vitamins, while parents may receive parental coaching. Child health clinics may be part of some curative care, community center or stand-alone places in an urban or rural area [15] In India, a community resource center was set up as a randomized controlled trial for women and children in informal settlements in the city. At the clinic, services were provided such as day care for malnourished children, community events to increase health literacy, group meetings to address women and children’s health concerns, and integrated home visits. After 2 years, child health outcomes improved and the concept had the potential to scale-up [29]

#### Access to essential medicines and interventions

Across service delivery systems, a range of interventions are provided from disease prevention, assessment and monitoring of health, growth, and development, to behavioral and social interventions. Disease prevention includes interventions such as with immunization of one or more vaccines [22, 32, 37–41], micronutrient supplements [40, 42], and insecticide nets [43]. Assessment and monitoring of the children occurs through both screening programs for nutritional deficiencies such as anemia [37, 44], or acute malnutrition [22], sensory losses including vision [38] and hearing [41], growth monitoring [32, 41], and specific diseases such as tuberculosis [44]. Behavioural and social interventions include both mother-child specific with breastfeeding counselling [26, 27, 32, 45, 46], and educational support such as the focus on improvement of parental knowledge of signs of illness [29, 32, 47], family planning [27, 30], and nutritional advice [27, 30, 32].

#### Healthy workforce

The systems of well-care for children are delivered by a workforce with various backgrounds depending on the type of service delivery as well as regional differences. During child health days, child health volunteers or child health workers are often the main providers of care [48–51]. The backgrounds of the providers vary, from those with previous training and work experience to new staff who receive training before the system of preventive health services, such as child health days, is implemented [30]. In middle- to-high-income countries, the workforce usually has a background in nursing [12, 26, 52–55] or medicine [12, 56, 57] with the latter encompassing different medical specializations, such as pediatrics [56, 57], and family medicine [57].

#### Government and leadership

The governance and leadership vary across the various systems of well-care visits for children. These systems can be permanent (governed by a local or state authority) [35, 58], mobile clinics [59], or temporary as part of a project [16, 29, 30]. Projects may involve the setting upp of separate structure [30], or be interventions within existing structures [60–63]. Governments may initiate integration, such as in Sierra Leone where six interventions were integrated into a program at established clinics to improve uptake of services [64]^,^ or researcher teams may run studies such as randomized control trials [23, 30, 65].

#### Funding

Well-care for children is funded in various formats: government funds, public-private insurances, research grants and user fees. Long-term funding for clinics is important for stability and a lack thereof may result in periodic closures [66]. In Laos, some clinics were partially dependent on non-governmental funds and areas had to rely on staff to accept retrospective payment or close down until government funding arrived/returned [66]. External funding may be needed to avoid the cost-barrier [59]. On an individual level, user fees are sometimes part of the funding for the well-care systems [67].

#### Health information systems

The systems report both digital and analogue methods to gather data specific to the child and the family, monitor population data and enhance communication between the leadership, such as the program director or health care worker, and the families. To track data and support workers on the services, digital solutions have been implemented ^[30,68]^, such as smart-phone reporting system [30, 68]. In other locations analogue methods are employed, such as through booklets [27, 69, 70] and flowsheets [71].

### Implementation strategies for well-care visits

The literature underscored the importance of implementation strategies to address barriers and enablers of well-care visits.

Structural aspects of the location of the well-care visits matter for uptake. Firstly, the geographical area for the system, whether for home-visits or clinics can be barriers for both attendees and health care providers [49, 72–75]. Across a relatively small geographical area, the actual availability of integrated care can vary from full-availability to limited [40, 76, 77]. From clinics in Kenya [78] and Malawi [49] to the United States of America [79], distances to clinics hinder attendees from taking advantage of integrated preventive care [78, 79]. In certain contexts, those with a regular place of care were two and a half more likely to have a well-baby checkup within 12 months than those without [80]. To overcome geographical barriers, the location of the clinic is important to address; a clinic on a community level can improve care [81] and mobile clinics can improve attendance [59]. Secondly, the actual layout of the clinic matters: if interventions are delivered by different health care workers in separate rooms which the family has to transition between during a visit, then there is a risk that not all interventions reach the family [77].

Moreover, the structure of the government and health policy influences the implementation strategy of well-care visits. In both LMIC and HIC, governments are important for coordination and strength of existing policies and its functionality is affected by corruption, political will, and economic and international policies [82]. Moreover, the functionality of sub-national governance is important [83]. In certain contexts, rural areas were found to have a weaker and more scattered central leadership working as enablers to implement systems of well-care visits [50]. Ultimately, partnerships among multiple stakeholders minimize duplication and improve coordination of care for the children [24].

Collaboration with existing governmental structures may enhance implementation of well-care visits. Building on existing structures has been successful for projects longevity [60–63]. Scaling up involves progress over time, with coordination and strengthening of existing structures with both human resource and capacity development [60, 62, 63]. Nonetheless, randomized control trials can deliver successful results but their generalizability to a real world setting in society has been questioned [84]. A review of randomized control trials suggests that these need to be replicated before policy implementation. In pilot programs, the participants engagement, retention, and adaptive behaviour need to be monitored to ensure realeffectiveness [84].

The transition from one intervention to multiple in the form of well-care visits may result in inadequately trained staff for particular interventions. In Gujarat State in India, immunization skills were found to be of high quality across staff, but the quality of other postnatal care services varied in the team [51]. In a review of a service aiming to promote correct breastfeeding practices among women through integration with other services, one clinic had 50% completion rate compared with other clinics that reached over 90% [85]. Even if professional development exists, barriers such as staff shortages result in a lack of time for sufficient training [56] and capacity to only provide services for some [64, 86].

Moreover, addressing socioeconomic differences within a given context may be a successful intervention strategy to enable uptake. Cash incentives have been found to improve attendance and compliance to the services, particularly in low resource settings [25, 34, 87, 88]. In an area where over 75% of the population lived in absolute poverty, cash payments had a large impact on health check-ups with timely starts of both immunization and growth monitoring, yet little effect on the 10 days check-in [87]. However, not all interventions seem to respond the same to cash incentives; an interventional study across 144 facilities showed no change in immunization, but well-care visits increased by 56% (child < 23 months) and 132% (child 24-59 months) respectively [34].

To communicate effectively, it is critical to language barriers are critical to address between parents and health care professionals during well-care visits. Infants with mother tongues other than the official languages are less likely to receive all recommended well-care visits [89–91]. Language barriers are identified among several minority groups, such as Puerto Rican children [91] and Bedouin children, respectively [74]. To counter the barriers services can be complemented with translators in-person [92] or via telephone [93] along with enhanced collaboration with the local community to reduce language obstacles [59].

Information strategies have attempted to improve attendance of well-care visits, in particular, which are related to low education and low income are associated with reduced attendance of integrated care. In some contexts, households with higher incomes had almost a 50% increase in odds of attending the clinic [94], while in others no direct effect on attendance was seen [95]. Lower educational level of the mother is associated with both reduced uptake and compliance [70, 96–98]. For women with incomplete elementary school there was almost four times higher risk of inadequate reach compared to those with higher educational backgrounds [96].

To improve health literacy, various informational strategies various methods have been tried, including calendars [99], postcards [97], and phone services [100, 101]. However, the knowledge of the content in the booklet varies between families [70, 88, 102]. Studies in both Kenya [102] and Nigeria [70] report only 1/5 of the families to have good knowledge of the contents of the booklets [70, 102]. In Burkina Faso, additional reminders through phone-calls to parents of high-risk infants raised attendance from 47 to 67% [100], however in other contexts the method made no significant difference. Interventions to improve the usage may increase uptake of the planned components of well-care visits [71].

### Potential child health effects of integrated systems

Benefits of integration of components occur at the individual, clinical and societal level. Concurrent delivery of interventions in a health care system offers an opportunity to amplify the effect of other services to maximise efficiency [28, 40, 103]. Integration enhances uptake and compliance of services in both HIC and LMIC, respectively [96, 103–106], and reduces the health care costs for governments [61, 63]. For example, immunization often has a high uptake and offers a possibility to deliver other essential services concurrently, enhancing uptake of “less-uptake services”, such as bed-net distribution and Vitamin A supplements [42]. The delivery of multiple interventions under a free health care service has been a critical instrument to reduce child mortality in Nepal [107] and health inequities in other countries [61]. Likewise, in locations without integration, there are missed opportunities to reach children [108] and areas with no or partial integration report higher mortality rates [109].

## Discussion

This scoping review aimed to map the existing literature on systems of well-care visits, identifying key components, implementation strategies, and potential impacts. Our findings, synthesized into a conceptual framework, highlight the fundamental pillars of systems of well-care visits, including essential interventions, workforce composition, and governance structures. Additionally, we identified key implementation strategies such as the integration of mHealth technologies and the scaling up of existing service delivery models. The potential impact of these strategies includes reduced healthcare costs, improved attendance rates, and enhanced uptake of preventive services, all of which contribute to addressing child health inequities and advancing the realization of the child health targets of the 2030 Agenda [110].

Integrated preventive child health care is a heterogenous field lacking in consensus on what is included, as already seen in the variation of terms used ranging from well-care visits [100, 111], and integrated child development services [40] to broader terms such as child health services [51, 112], and systems of well-care visits [9]. Similar variety has been reported in child health before, such as in the field of pediatrics in Europe where the age range varies, from 18 or 19 years to 14 years or younger, depending on the country [13]. To enhance collaboration and future research and policy efforts, larger consensus on how to describe systems of well-care visits would improve the synthesis of the field.

Our results show that the number of visits varies, in some places far below the new guidelines by the WHO, which recommends 13 visits before the age of 5, and others are above [9]. These results are similar to the findings of Wood and Blair’s review on the trends in child health visits in HIC from a decade back [12]. The wide span of visits (3–18) within the different programs found in Wood and Blair’s review [12] can, according to our review, be expanded to ensure pediatric populations across geographical and economic contexts are reached with all the recommended components of well-care visits [9].

Furthermore, our findings show a more diverse range of interventions provided compared to the Wood and Blair’s review [12] of HIC settings only. where all locations included physical examination, newborn blood screening, universal neonatal hearing screening, and vision screening [12]. The results indicate that in many contexts systems only include a few of the components of the comprehensive program recommended by the WHO and UNICEF [9]. There are potent barriers on an individual, health facility, and societal level (Fig. 2) that needs to be addressed to ensure the desired components of well-care visits are both provided for and received by all children globally.

In this review, where low-income countries were included, we found numerous studies supporting the potential of health care workers with various education levels, including volunteers [47–49, 75, 113–115], to provide the components of well-care for children. This is a discrepancy from previous reviews which have reported nurses and physicians as the main providers of systems of well-care visits, which is likely a reflection of the extended scope of this review covering health care systems from various resource settings. The results suggest that for systems of well-care visits deficits in human resources may be improved through task-shifting as the recent guidelines from WHO and UNICEF also underscore [9]^.^

Our results highlight the importance of the government involvement for systems of well-care visits to remain over time. Systems that are integrated within existing structures seem to remain longer, indicating that further work on improving well-care visits should be done in close collaboration with existing structures [72, 74, 75]. Moreover, the systems of well-care visits seem to not only be a useful approach in middle- and high-income countries, but low-income countries as well, supporting the need for further work to implement systems of well-care visits across income settings. With existing guidelines now covering up to adolescent years [9], there is a need to understand the existing models from 5 to 18 years of age.

Barriers faced within systems of well-care visits share key themes across settings, and a variety of solutions have been explored to overcome these. Community engagement, health care workers and governments all play a critical role for systems to establish themselves effectively and to persist over time [116]. For well-care visits to be a default, a multisectoral approach is critical to respond to the various socioeconomic factors that affect and may increase in importance as climate change, wars, and other factors disturb our existing structures [116].

Our study highlights clear knowledge gaps in the existing literature. First, few articles detailed the long-term effects and outcomes of systems which makes it challenging to assess the impact and sustainability of systems of well-care visits. Second, there is a lack of understanding of how children and caregivers, other than mothers, experience the system and concurrent challenges; more than 90% of the articles focused on the mother of the child as opposed to fathers or grandparents. This is despite the fact that fathers and grandparents influence the primary care givers’ decisions regarding the child’s health and sometimes act as gatekeepers for accessing care [117]. Third, despite the majority of child mortality and morbidity burden being in low- or lower middle-income countries, most original studies and articles included in this review are from upper middle-income and high-income countries. Fourth, since the methods varied significantly across studies and quantitative comparable measures specifically linked to preventive care were often lacking, no general comparable assessments of different systems of preventive child health care were possible.

This scoping review showcases the wide variety and quantity of both primary articles and reviews that were included through a systematic search. However, the synthesis of such large material brings some inherent limitations to the study. First, the limitations of languages and timeframe are likely to exclude some relevant articles. Second, the screening and data extraction were done by one person, introducing a risk for bias, even if 10% were double-checked by two other authors with no major discrepancies. Thirdly, due to the scope of the review, a quality assessment of the articles was not deemed possible. Fourthly, this study focused on peer-reviewed literature, and some systems of well-care visits and programs are likely described in grey literature and thus not included. Nonetheless, the scoping review covers a range of material from low- to high resource settings and maps clear knowledge and gaps thereof synthesizing evidence in a scattered field.

## Conclusions

This scoping review synthesizes current evidence on systems of well-care visits, outlining their core components, implementation strategies, and potential impacts on child health outcomes. For decades numerous landmark reports have called for integrated approaches to reduce child morbidity and mortality. A rich plethora of solutions have been developed to deliver preventive child health services, often facing similar barriers. To accelerate the implementation of cost-effective, robust, and long-lasting integrated preventive child health care approaches, there is a need to understand and learn from the different methods to continuously develop context-sensitive variants of preventive integrated child health systems, such as well-care visits. Addressing the research gaps described above may prove pivotal in ensuring good health and well-being for all children.

## Supplementary Information

Supplementary Material 1: Additional file with the detailed literature search, list of variables used in data chart, PRISMA-ScR Checklist, and all the documents included in the review.

Supplementary Material 2: Additional file with the populated version of the data extraction sheet used.

## Data Availability

All articles analyzed during the study are described in the attached supplementary materials. Table 1 and Fig. 2 were developed based on a data extraction chart of the included articles, which can be found in the attached file to the article (Supplementary material 2).

## Abbreviations

AN: Alma Nordenstam
DH: Daniel Helldén
IMCI: Integrated Management of Childhood Illnesses
LMIC: Low- and middle-income countries
MDGs: Millennium Development Goals
OB: Olivia Biermann
SDGs: Sustainable Development Goals
TA: Tobias Alfvén
AE: Anna-Theresia Ekman
