# Supplementary material for: Framework for systems of pediatric well-care visits: a scoping review
Source: BMC Glob Public Health. 2025 Nov 13;3:100. doi: 10.1186/s44263-025-00211-4 (PMC12613775; doi:10.1186/s44263-025-00211-4)
Supplement: Supplementary file 1 — Supplementary Material 1: Additional file with the detailed literature search, list of variables used in data chart, PRISMA-ScR Checklist, and all the documents included in the review. [file 44263_2025_211_MOESM1_ESM.pdf]

Supplementary Material 1

Framework for Systems of Pediatric Well-Care Visits: a Scoping Review

Authors: Nordenstam, A., Helldén, D, Ekman, A-T, Blennow, M., Ndeezi, G., Biermann, O., Alfvén, T.

Detailed literature search strategy…………………………………………………...….2

List of variables used in data chart……………………………………...……………..5

PRISMA-ScR Checklist………………………………………………………………..7

All documents included in the review………………………………………………...…9

Detailed literature search strategy

Relevant articles were identified through the most relevant databases for the study: PubMed, Cochrane Library, Web of Science and OVID Global Health. Please note that separate components of preventive child health care, including immunization programs, screenings and home visits were not searched for directly given the system-focus of the study.

1. PubMed

Concept 1: Child Health Services

Key words:

”child health service*”[Title/Abstract] OR “infant health service*”[Title/Abstract] ”maternal-child health center*”[Title/Abstract] OR “preventive health service*”[Title/Abstract] OR “preventive health program*”[Title/Abstract]

MeSH:

”child health services”[MeSH Terms] OR “maternal-child health centers”[MeSH Terms]

Combined:

”child health service*”[Title/Abstract] OR “infant health service*”[Title/Abstract] OR ”maternal-child health center*”[Title/Abstract] OR “preventive health service*”[Title/Abstract] OR “preventive health program*”[Title/Abstract] OR ”child health services”[MeSH Terms] OR “maternal-child health centers”[MeSH Terms]

Concept 2: Children under 5

Key words: "infan*"[Title/Abstract] OR "preschool child*"[Title/Abstract] OR “newborn”[Title/Abstract]

MeSH: (infant[MeSH Terms]) OR (child, preschool[MeSH Terms])

Combined: "infan*"[Title/Abstract] OR "preschool child*"[Title/Abstract] OR “newborn”[Title/Abstract] OR ”infant”[MeSH Terms] OR ”child, preschool”[MeSH Terms]

Concept 3: Avoid curative care

MeSH: NOT (hospitalization[MeSH Terms]))

Concept 4: Limit articles to 2000-2021

MeSH: 2000/01/01:2021/12/31[Date - Publication]

Concept 5: Limit articles to English

MeSH: english[Filter]

Search Query: (((("child health service*"[Title/Abstract] OR "infant health service*"[Title/Abstract] OR "maternal child health center*"[Title/Abstract] OR "child health services"[MeSH Terms] OR "preventive health service*"[Title/Abstract] OR "maternal-child health centers"[MeSH Terms]) AND 2000/01/01:2021/12/31[Date - Publication]) NOT ("hospitalization"[MeSH Terms] AND "english"[Language])) AND ("infan*"[Title/Abstract] OR "preschool child*"[Title/Abstract] OR "infant"[MeSH Terms] OR "child, preschool"[MeSH Terms]) AND "english"[Language]) AND (english[Filter])

Results: 7916

2. Cochrane Library

Concept 1:

Key words (child health service*):ti,ab,kw OR (infant health service*):ti,ab,kw OR (preventive health service*):ti,ab,kw OR (preventive health program*):ti,ab,kw OR (maternal child health center*):ti,ab,kw

MeSH OR MeSH descriptor: [Child Health Services] explode all trees OR MeSH descriptor: [Maternal-Child Health Centers] explode all trees

Concept 2:

Key words: (infan*):ti,ab,kw OR (“preschool child”*):ti,ab,kw OR (newborn):ti,ab,kw
MeSH OR MeSH descriptor: [Infant] explode all trees OR MeSH descriptor: [Child, Preschool] explode all trees

Concept 3:

MeSH NOT MeSH descriptor: [Hospitalization] in all MeSH products

Search query: ((child health service*):ti,ab,kw OR (infant health service*):ti,ab,kw OR (preventive health service*):ti,ab,kw OR (preventive health program*):ti,ab,kw OR (maternal child health center*):ti,ab,kw OR MeSH descriptor: [Child Health Services] explode all trees OR MeSH descriptor: [Maternal-Child Health Centers] explode all trees) AND ((infan*):ti,ab,kw OR (“preschool child”*):ti,ab,kw OR (newborn):ti,ab,kw OR MeSH descriptor: [Infant] explode all trees OR MeSH descriptor: [Child, Preschool] explode all trees) NOT (MeSH descriptor: [Hospitalization] in all MeSH products)

Filter: 2000-2021

Results: 920

3. Web of Science

Concept 1:

Keywords: (((((((TS=(”child health service*”)) OR TS=(”preventive health service*”)) OR TS=(”continuity of patient care”)) OR TS=(”maternal child health center*”)) OR TS=(“infant health service*”)) OR TS=(“preventive health program*”)) OR TS=(”maternal-child health center*”))

Concept 2:

Key words: (TS=(infan*)) OR TS=(preschool child*) OR TS=(newborn)

Search query: ((((((((TS=("child health
service*")) OR TS=("preventive health service*")) OR TS=("continuity of patient care")) OR TS=("maternal child health center*")) OR TS=("infant health service*")) OR TS=("preventive hea

lth program*")) OR TS=("maternal-child health center*"))) *AND*
((TS=(infan*)) OR TS=(preschool child*) OR TS=(newborn))

Filter: 2000-2021, English

Results: 483

4. OVID Global Health

Concept 1:
Key words: "child health service*".ab,ti. OR "infant health service* ".ab,ti. OR"preventive health program* ".ab,ti. OR "preventive health service*.ab,ti. OR "maternal-child health center* ".ab,ti.

Concept 2
Key words: infan*.ab,ti. OR newborn.ab,ti. OR "preschool child*".ab,ti. OR newborn.ab,ti.

Search query: ("child health service*".ab,ti. OR "infant health service* ".ab,ti. OR"preventive health program* ".ab,ti. OR "preventive health service*.ab,ti. OR "maternal-child health center* ".ab,ti.) AND (infan*.ab,ti. OR newborn.ab,ti. OR "preschool child*".ab,ti. OR newborn.ab,ti.)

Filter: 2000-2021

Results: 205

List of variables used in data chart

1.Screening
Full-text available: available through the data bases or directly from the journal
English language: available in full-text, not only abstract, in the English language *Primary article or review*: excluding all case reports, letters, government reports, non-peer reviewed literature etc.
System: included at least two interventions delivered at the same place and time
Prevention: all interventions are preventive in the described system, if preventive and curative

care were combined in any system they were excluded , if an article included two systems, one preventive and one curative, and they were delivered separately and had separate results, the article was included
Children under five: interventions for children before their fifth birthday. Excludes articles which included only age 5+, combined 5+ and under, or undisclosed or unclear age group

2. General information
Authors: as stated in the journal article
Type of document: primary article or review
Journal: as stated in the journal article
Year of publication: as stated in the journal article

3. Study description
Country/countries: country extracted based on geographical information in the article. If country was not named, the country was extracted based on the geographical location stated and the year of the study
World bank income level: derived from world bank classification based on the year of publication
Level of society: community/city/county/national/international
Year(s) of study: as stated in the journal article
Setting: urban/rural
Study group number: number of participants as stated in the journal article
Study group age(range): as stated in the journal article
Sex of study group: female/male/other
Study design: quantitative/qualitativ/mixed

4. Health systems framework
Service delivery: location of the system (at home/health facility permanent or temporary/mobile clinic etc), number of facilities, service quality, architectural considerations of the facility for system-level etc
Health Workforce: type of workforce (volunteers, child health workers, nurses, doctors etc), challenges/opportunities for the workforce to deliver system-based well-care visits
Health information: how/what/when data is monitored, type of data, method of tracking and storage (digital/analogue), information at a parental level/ health facility/governmental level
Essential medicines: type of interventions delivered, access to the material to provide those, how/what/when interventions are delivered etc.
Health Financing: type of funding (out-of-pocket/aid/research-grant/governmental), longevity of the funding (temporary/permanent funding), changes in funding etc.
Leadership and Governance: level of leadership (health facility level/community/city/county/national/international), changes in leadership and governance etc.

5. Implementation
Barriers: any hinders to the system, either on individual (child/parent/provider), health facility, community or societal level
Enablers: any enablers to the system, either on individual (child/parent/provider), health facility, community or societal level

PRISMA-ScR Checklist

SECTION	ITEM	PRISMA-ScR CHECKLIST ITEM	REPORTED ON PAGE #
**TITLE**			
Title	1	Identify the report as a scoping review.	1
**ABSTRACT**			
Structured summary	2	Provide a structured summary that includes (as applicable): background, objectives, eligibility criteria, sources of evidence, charting methods, results, and conclusions that relate to the review questions and objectives.	2
**INTRODUCTION**			
Rationale	3	Describe the rationale for the review in the context of what is already known. Explain why the review	2-3

SECTION	ITEM	PRISMA-ScR CHECKLIST ITEM	REPORTED ON PAGE #
		questions/objectives lend themselves to a scoping review approach.	
Objectives	4	Provide an explicit statement of the questions and objectives being addressed with reference to their key elements (e.g., population or participants, concepts, and context) or other relevant key elements used to conceptualize the review questions and/or objectives.	2-3
**METHODS**			
Protocol and registration	5	Indicate whether a review protocol exists; state if and where it can be accessed (e.g., a Web address); and if available, provide registration information, including the registration number.	3
Eligibility criteria	6	Specify characteristics of the sources of evidence used as eligibility criteria (e.g., years considered, language, and publication status), and provide a rationale.	3
Information sources*	7	Describe all information sources in the search (e.g., databases with dates of coverage and contact with authors to identify additional sources), as well as the date the most recent search was executed.	3
Search	8	Present the full electronic search strategy for at least 1 database, including any limits used, such that it could be repeated.	3
Selection of sources of evidence†	9	State the process for selecting sources of evidence (i.e., screening and eligibility) included in the scoping review.	3
Data charting process‡	10	Describe the methods of charting data from the included sources of evidence (e.g., calibrated forms or forms that have been tested by the team before their use, and whether data charting was done independently or in duplicate) and any processes for obtaining and confirming data from investigators.	3-4
Data items	11	List and define all variables for which data were sought and any assumptions and simplifications made.	3, Appendix
Critical appraisal of individual sources of evidence§	12	If done, provide a rationale for conducting a critical appraisal of included sources of evidence; describe the methods used and how this information was used in any data synthesis (if appropriate).	-
Synthesis of results	13	Describe the methods of handling and summarizing the data that were charted.	3-4
**RESULTS**			
Selection of sources of evidence	14	Give numbers of sources of evidence screened, assessed for eligibility, and included in the review, with reasons for exclusions at each stage, ideally using a flow diagram.	3
Characteristics of sources of evidence	15	For each source of evidence, present characteristics for which data were charted and provide the citations.	Appendix
Critical appraisal within sources of evidence	16	If done, present data on critical appraisal of included sources of evidence (see item 12).	No
Results of individual sources of evidence	17	For each included source of evidence, present the relevant data that were charted that relate to the review questions and objectives.	Appendix
Synthesis of results	18	Summarize and/or present the charting results as they relate to the review questions and objectives.	4-10
**DISCUSSION**			
Summary of evidence	19	Summarize the main results (including an overview of concepts, themes, and types of evidence available), link to	10-11

SECTION	ITEM	PRISMA-ScR CHECKLIST ITEM	REPORTED ON PAGE #
		the review questions and objectives, and consider the relevance to key groups.	
Limitations	20	Discuss the limitations of the scoping review process.	11
Conclusions	21	Provide a general interpretation of the results with respect to the review questions and objectives, as well as potential implications and/or next steps.	11
**FUNDING**			
Funding	22	Describe sources of funding for the included sources of evidence, as well as sources of funding for the scoping review. Describe the role of the funders of the scoping review.	3

Preferred Reporting Items for Systematic reviews and Meta-Analyses extension for Scoping Reviews (PRISMA-ScR) Checklist

JBI = Joanna Briggs Institute; PRISMA-ScR = Preferred Reporting Items for Systematic reviews and Meta-Analyses extension for Scoping Reviews.

* Where *sources of evidence* (see second footnote) are compiled from, such as bibliographic databases, social media platforms, and Web sites.

† A more inclusive/heterogeneous term used to account for the different types of evidence or data sources (e.g., quantitative and/or qualitative research, expert opinion, and policy documents) that may be eligible in a scoping review as opposed to only studies. This is not to be confused with *information sources* (see first footnote).

‡ The frameworks by Arksey and O'Malley (6) and Levac and colleagues (7) and the JBI guidance (4, 5) refer to the process of data extraction in a scoping review as data charting.

§ The process of systematically examining research evidence to assess its validity, results, and relevance before using it to inform a decision. This term is used for items 12 and 19 instead of "risk of bias" (which is more applicable to systematic reviews of interventions) to include and acknowledge the various sources of evidence that may be used in a scoping review (e.g., quantitative and/or qualitative research, expert opinion, and policy document).

From: Tricco AC, Lillie E, Zarin W, O'Brien KK, Colquhoun H, Levac D, et al. PRISMA Extension for Scoping Reviews (PRISMAScR): Checklist and Explanation. Ann Intern Med. 2018;169:467–473. doi: 10.7326/M18-0850.

All documents included.

Organised by type of document followed by income-level of the setting.

**Primary articles**							
**Title**	**Authors**	**Journal/Publisher**	**Year**	**Country/Countries**	**World Bank Income Level**	**Setting: urban, rural, both**	**Study design: quantitative, qualitative, mixed**
**High income countries**							
Three-year follow-up of a	Taylor RW,	BMC Public Health	BMC	New Zealand	H	Urban	Quantitative

randomised controlled trial to reduce excessive weight gain in the first two years of life: protocol for the POI follow-up study.	Heath AL, Galland BC, Cameron SL, Lawrence JA, Gray AR, et al.		Pub lic Hea lth				
Assessing the quality of preschool child health surveillance in primary care: a pilot study in one health district.	Hampshir e AJ, Blair ME, Crown NS, Avery AJ, Williams EI.	Child Care Health Dev.	200 2	Unted KIngdom	H	Urba n	Mixed
The effect of Taiwan's National Health Insurance on infants' preventive care use and inpatient care use.	Chen C-S, Liu T-C, Lin H-C, Tian W-H.	Health Policy	200 7	Taiwan	H	Both	Quantita tive
Poverty, insurance, and well-baby care among mainland Puerto Rican children.	Gorman BK, Landale NS, Oropesa RS.	Soc Biol.	200 1	United States	H	Both	Mixed
A feasibility study to improve practice nurses' competence and confidence in providing care for mothers and infants.	Bogossia n F, Brodribb W, Farley R, Goodwin H, Tin A, Young J.	Contemp Nurse.	201 7	Australia	H	Urba n	Quantita tive
A health passport for adolescent	Stevens-Simon C,	Stevens-Simon C,	200 1	United States	H		Mixed

parents and their children.	Kelly L, Brayden RM.	Kelly L, Brayden RM.					
The association of family continuity with infant health service use.	Clark EC, Saultz J, Buckley DI, Rdesinski R, Goldberg B, Gill JM.	J Am Board Fam Med.	200 8	United States	H		Quantut ative
Effectiveness of an early intervention on infant feeding practices and "tummy time": a randomized controlled trial.	Wen LM, Baur LA, Simpson JM, Rissel C, Flood VM.	Arch Pediatr Adolesc Med.	201 1	Australia	H	Urba n	Mixed
Design and Operation of the Transformed National Healthy Start Evaluation.	Banks JE, Dwyer M, Hirai A, Ghandour RM, Atrash HK.	Matern Child Health J.	201 7	United States	H	Both	Quantita tive
Does trainee involvement affect anticipatory guidance in well-child care?	Sgrignoli NR, Lehman EB, Sekhar DL	Clin Pediatr (Phila).	201 4	United States	H	Urba n	Quantita tive
Practice based education to improve delivery systems for prevention in primary care: randomised trial.	Margolis PA, Lannon CM, Stuart JM, Fried BJ, Keyes-Elstein L, Moore DE.	BMJ	200 4	United States	H	Urba n	Quantita tive

The Rourke Baby Record Infant/Child Maintenance Guide: do doctors use it, do they find it useful, and does using it improve their well-baby visit records?	Rourke L, Godwin M, Rourke J, Pearce S, Bean J	BMC Fam Pract.	2009	Canada	H	Urban	Quantitative
Identifying Continuous Quality Improvement Priorities in Maternal, Infant, and Early Childhood Home Visiting.	Preskitt J, Fifolt M, Ginter PM, Rucks A, Wingate MS.	J Public Health Manag Pract.	2016	United States	H	Both	Quantitative
The effects of an area-based intervention on the uptake of maternal and child health assessments in Australia: a community trial.	Kelaher M, Dunt D, Feldman P, Nolan A, Raban B	BMC Health Serv Res.	2009	Australia	H	Both	Quantitative
Impact of a decline in Colorado Medicaid managed care enrollment on access and quality of preventive primary care services.	Berman S, Armon C, Todd J.	Pediatrics	2005	United States	H	Both	Quantitative

Evaluation of the Implementation of the Healthy Start Program: Findings from the 2016 National Healthy Start Program Survey.	Parasuraman SR, de la Cruz D.	Matern Child Health J.	2019	United States	H	Both	Quantitative
Improving preventive service delivery through office systems.	Bordley WC, Margolis PA, Stuart J, Lannon C, Keyes L.	Pediatrics.	2001	United States	H	Urban	Quantitative
Women with non-Swedish speaking background and their children: a longitudinal study of uptake of care and maternal and child health.	Fabian H, Rådestad I, Rodriguez A, Waldenström U.	Acta Paediatr.	2008	Sweden	H	Both	Quantitative
Effects of fully-established Sure Start Local Programmes on 3-year-old children and their families living in England: a quasi-experimental observational study.	Melhuish E, Belsky J, Leyland AH, Barnes J.	Lancet	2008	England	H	Both	Quantitative
Parents with low literacy report higher quality of parent-provider	Rosenthal MS, Socolar RR, DeWalt	Ambul Pediatr.	2007	United States	H	Urban	Mixed

relationships in a residency clinic.	DA, Pignone M, Garrett J, Margolis PA.						
Variation in community intervention programmes and consequences for children and families: the example of Sure Start Local Programmes	Melhuish E, Belsky J, Anning A, Ball M, Barnes J, Romaniuk H, et al.	J Child Psychol Psychiatry.	2007	England	H	Both	Quantitative
Assessing the impact of pediatric-based development services on infants, families, and clinicians: challenges to evaluating the Health Steps Program.	Guyer B, Hughart N, Strobino D, Jones A, Scharfstein D.	Pediatrics	2000	United States	H	Both	Mixed
Electronic health record use and preventive counseling for US children and adolescents.	Rand CM, Blumkin A, Szilagyi PG.	J Am Med Inform Assoc	2014	United States	H	Both	Quantitative
Impact of intimate partner violence on children's well-child care and medical home.	Bair-Merritt MH, Crowne SS, Burrell L, Caldera D, Cheng TL,	Pediatrics	2008	United States	H	Both	Mixed

	Duggan AK.						
Receipt of routine preventive care among infant daughters and sons of immigrant mothe rs in Ontario, Canada: a retrospective cohort study.	Pulver A, Guttman n A, Ray JG, O'Campo PJ, Urquia ML.	BMJ Open.	202 0	Canada	H	Both	Quantita tive
Satisfaction with health care for young children. Pediatrics.	Halfon N, Inkelas M, Mistry R, Olson LM.	Pediatrics	200 4	United States	H	Both	Quantita tive
Quality in preventive and health-promoting services: constructing an understanding th rough process.	Hanafin S, Cowley S.	J Nurs Manag	200 6	Ireland	H	Both	Quantita tive
Parent-directed intervention in promoting knowledge of pediatric nutrition and healthy lifestyle among low-SES families with toddlers: A randomized controlled trial.	LoRe D, Leung CYY, Brenner L, Suskind DL.	Child Care Health Dev.	201 9	United States	H	Urba n	Quantita tive
Health needs assessment and the ecology of	Trout F.	Aust Health Rev.	200 1	New Zealand	H	Both	Mixed

care: a research note.							
An intervention for reducing secondary traumatization and improving professional self-efficacy in well baby clinic nurses following war and terror: a random control group trial.	Berger R, Gelkopf M.	Int J Nurs Stud.	2011	Israel	H	Both	Quantitative
Supporting maternal transition: continuity, coaching, and control.	Rowe J, Barnes M, Sutherns S.	J Perinat Educ.	2013	Australia	H	Both	Mixed
New opportunities for integrated child health systems: results from the multifaceted pre-to-three program.	Cuellar AE, Wagner TH, Hu T-W, Peifer K, Kitzman H, Tobin SJ, et al.	Am J Public Health	2003	United States	H	Both	Quantitative
Health needs and timeliness of assessment of Victorian children entering out-of-home care: An audit of a multidisciplinary assessment clinic.	McLean K, Little K, Hiscock H, Scott D, Goldfeld S.	J Paediatr Child Health.	2019	Australia	H	Urban	Quantitative

WIC participation, breastfeeding practices, and well-child care among unmarried, low-income mothers.	Chatterji P, Brooks-Gunn J.	Am J Public Health.	2004	United States	H	Urban	Quantitative
Successful early postpartum support linked to management, informational, and relational continuity.	Barimani M, Vikström A.	Midwifery.	2015	Sweden	H	Urban	Qualitative
The perceptions of statutory service providers of a local Sure Start programme: a shared agenda?	Edgley A, Avis M.	Health Soc Care Community.	2007	United Kingdom	H	Both	Qualitative
Effects of continuity of care in infancy on receipt of lead, anemia, and tuberculosis screening.	Flores AI, Bilker WB, Alessandrini EA.	Pediatrics	2008	United States	H	Both	Quantitative
Maternal and Infant Services: Examination of access in a culturally diverse community.	Comino EJ, Harris E.	J Paediatr Child Health.	2003	Australia	H	Both	Quantitative
Primary language of parent is associated with disparities in pediatric preventive care.	Cohen AL, Christakis DA.	J Pediatr.	2006	United States	H	Both	Quantitative

Statewide quality improvement outreach improves preventive services for young children.	Shaw JS, Wasserman RC, Barry S, Delaney T, Duncan P, Davis W, et al.	Pediatrics.	2006	Canada	H	Both	Quantitative
A Stepped Intervention Increases Well-Child Care and Immunization Rates in a Disadvantaged Population.	Hambidge SJ, Phibbs SL, Chandramouli V, Fairclough D, Steiner JF.	Pediatrics.	2009	United States	H	Urban	Quantitative
Well child care in the United States: Racial differences in compliance with guidelines.	Ronsaville DS, Hakim RB.	Am J Public Health.	2000	United States	H	Both	Quantitative
From concept to application: The impact of a community-wide intervention to improve the delivery of preventive services to children. Pediatrics.	Margolis PA, Stevens R, Bordley WC, Stuart J, Harlan C, Keyes-Elstein L, et al.	Pediatrics.	2001	United States	H	Urban	Mixed
Utilization of child health services, stress, social support and child characteristics in	Lagerberg D, Magnusson M.	Scand J Public Health	2013	Sweden	H	Both	Quantitative

primiparous and multiparous mothers of 18-month-old children.							
Usage, adherence and attrition: how new mothers engage with a nurse-moderated web-based intervention to support maternal and infant health. A 9-month observational study.	Sawyer MG, Reece CE, Bowering K, Jeffs D, Sawyer ACP, Peters JD, et al.	BMJ Open.	2016	Australia	H	Urban	Quantitative
Detection of child abuse by Dutch preventive child-healthcare doctors and nurses: Has it changed?	Reijneveld SA, de Meer G, Wiefferink CH, Crone MR.	Child Abus Negl	2008	Netherlands	H	Both	Quantitative
Comparing child health, access to care, and utilization of health services between Ohio Appalachia's River and non-river bordering counties.	Smith LH, Holloman C.	J Community Health.	2011	United States	H	Both	Quantitative
Effectiveness of the Comprehensive Childhood Protection System for	Arcos E, Muñoz LA, Sanchez X, Vollrath	Rev Lat Am Enfermagem.	2013	Chile	H	Urban	Mixed

vulnerable moth ers and children.	A, Gazmuri P, Baeza M.						
Narrowing the income gaps in preventive care for young children: Families in healthy steps.	McLearn KT, Strobino DM, Minkovitz CS, Marks E, Bishai D, Hou W.	J Urban Heal	200 4	United States	H	Both	Mixed
Effect of revised nursery orders on newborn preventive services.	Madlon-Kay DJ.	J Am Board Fam Med	201 1	United States	H	Urba n	Quantita tive
Expectations of the child health nurse in Sweden: two perspectives.	Fägerskiöl d A, Ek AC.	Int Nurs Rev	200 3	Sweden	H	Both	Qualitati ve
Satisfaction with child health services in the non-government sector of Hong Kong: Consumer evaluation.	Chan SS-C, Twinn S.	Nurs Heal Sci [Internet].	200 3	Hong Kong	H	Urba n	Quantita tive
Reassurance or judgement? Parents' views on the delivery of child health surveillance programmes.	Roche B, Cowley S, Salt N, Scammell A, Malone M, Savile P, et al.	Fam Pract [Internet].	200 5	United Kingdom	H	Urba n	Qualitati ve

How indigenous mothers experience selecting and using early childhood development services to care for their infants.	Wright AL, Jack SM, Ballantyne M, Gabel C, Bomberry R, Wahoush O.	Int J Qual Stud Health Well-being.	2019	Canada	H	Both	Qualitative
Postnatal care in low-income urban African American women: relationship to level of prenatal care sought.	York R, Tulman L, Brown K.	Perinatol [Internet].	2000	United States	H	Urban	Mixed
Strategies to improve immunization rates and well-child care in a disadvantaged population - A cluster randomized controlled trial.	Hambidge SJ, Davidson AJ, Phibbs SL, Chandramouli V, Zerbe G, LeBaron CW, et al.	Arch Pediatr Adolesc Med.	2004	United States	H	Urban	Quantitative
Health service use and costs for infant behaviour problems and maternal stress.	Le HND, Gold L, Mensah FK, Cook F, Bayer JK, Hiscock H.	J Paediatr Child Health.	2016	Australia	H	Urban	Quantitative
Parental expectations of maternal and	Bradt L, Vandenbroeck M, Lammert	Soc Work Public Health.	2015	Belgium	H	Urban	Mixed

child health services.	yn J, Bouverne -De Bie M.						
Practising proportionate universalism - a study protocol of an extended postnatal home visiting programme in a disadvantaged area in Stockholm, Sweden.	Burström B, Marttila A, Kulane A, Lindberg L, Burström K	BMC Health Serv Res.	2017	Sweden	H	Urban	Quantitative
Challenges in monitoring the development of young children in remote Aboriginal health services: clinical audit findings and recommendations for improving practice.	D'Aprano A, Silburn S, Johnston V, Bailie R, Mensah F, Oberklaid F, et al.	Rural Remote Health.	2016	Australia	H	Both	Quantitative
Ethnic and Indigenous access to early childhood healthcare services in Australia: parents' perceived unmet needs and related barriers.	Ou L, Chen J, Garrett P, Hillman K.	Aust N Z J Public Health.	2011	Australia	H	Both	Mixed
Adherence to Well-Child Care and Home Visiting	Goyal NK, Brown CM, Folger AT,	Matern Child Health J.	2020	United States	H	Urban	Quantitative

Enrollment Associated with Increased Emergency Department Utilization.	Hall ES, Van Ginkel JB, Ammerman RT.						
Medical Home-Head Start Partnership to Promote Early Learning for Low-Income Children.	Grant AR, Ebel BE, Osman N, Derby K, DiNovi C, Grow HM.	Health Promot Pract.	2019	United States	H	Urban	Quantitative
Maternal perceptions of social context and adherence to maternal and child health (MCH) clinic recommendations among marginalized Bedouin mothers.	Daoud N, Shoham-Vardi I.	Matern Child Health J.	2015	Israel	H	Both	Mixed
Characteristics, access, utilization, satisfaction, and outcomes of healthy start participants in eight sites.	Rosenbach M, O'Neil S, Cook B, Trebino L, Walker DK.	Matern Child Health J.	2010	United States	H	Both	Quantitative
Smoothing out the transition of care between maternity and child and family health services: perspectives of child and family	Psaila K, Kruske S, Fowler C, Homer C, Schmied V.	BMC Pregnancy Childbirth	2014	Australia	H	Both	Mixed

health nurses and midwives’.							
The Generation R Study: design and cohort update 2010.	Jaddoe VW V, van Duijn CM, van der Heijden AJ, Mackenbach JP, Moll HA, Steegers EAP, et al.	Eur J Epidemiol	2010	Netherlands	H	Urban	Quantitative
Immigrant parents’ experience with the Swedish child health care system: A qualitative study.	Mangrio E, Persson K.	BMC Fam Pract.	2017	Sweden	H	Urban	Qualitative
AmeriCorps Members increase enrollment in Medicaid/CHIP and preventive care utilization at a community health center.	Stipelman C, Dinkins CP, Pruhs A, Serr R, Young P.	J Health Care Poor Underserved.	2014	United States	H	Urban	Quantitative
How do Australian maternity and early childhood health services identify and respond to the settlement experience and	Yelland J, Riggs E, Wahidi S, Fouladi F, Casey S, Szwarc J, et al.	BMC Pregnancy Childbirth.	2014	Australia	H	Urban	Qualitative

social context of refugee background families?							
Group Well-Child Care and Health Services Utilization: A Bilingual Qualitative Analysis of Parents' Perspectives.	Oldfield BJ, Nogelo PF, Vázquez M, Ona Ayala K, Fenick AM, Rosenthal MS.	Matern Child Health J	2019	United States	H	Urban	Qualitative
Organizational differences in early child health care -- mothers' and nurses' experiences of the services.	Magnusson M, Lagerberg D, Sundelin C.	Scand J Caring Sci.	2010	Sweden	H	Both	Quantitative
Selected findings from the cross-site evaluation of the Federal Healthy Start Program.	Drayton VLC, Walker DK, Ball SW, Donahue SMA, Fink R V	Matern Child Health J.	2015	United States	H	Both	Quantitative
Area-Level Socioeconomic Factors Are Associated With Noncompletion of Pediatric Preventive Services.	Jones MN, Brown CM, Widener MJ, Sucharew HJ, Beck AF.	J Prim Care Community Health.	2016	United States	H	Urban	Quantitative

Access to preventive services after the integration of oral health care into early childhood education and medical care.	Burgette JM, Preisser JS, Rozier RG.	J Am Dent Assoc.	2018	United States	H	Both	Mixed
A case study of well child care visits at general practices in a region of disadvantage in Sydney.	Garg P, Eastwood J, Liaw S-T, Jalaludin B, Grace R	PLoS One.	2018	Australia	H	Urban	Mixed
General practitioner provision of preventive child health care: analysis of routine consultation data.	Wood R, Wilson P.	BMC Fam Pract	2012	United Kingdom	H	Both	Quantitative
Well-child care clinical practice redesign at a community health center: provider and staff perspectives.	Mooney K, Moreno C, Chung PJ, Elijah J, Coker TR.	J Prim Care Community Health.	2014	United States	H	Urban	Qualitative
Predictors of well child care adherence over time in a cohort of urban Medicaid-eligible infants.	Van Berckelaer AC, Mitra N, Pati S.	BMC Pediatr.	2011	United States	H	Urban	Quantitative

Formative research in the development of a salutogenic early intervention home visiting program integrated in public child health service in a multiethnic population in Norway.	Leirbakk MJ, Torper J, Engebretsen E, Opsahl JN, Zeanah P, Magnus JH.	BMC Health Serv Res.	2018	Norway	H	Rural	Qualitative
Development of a bundle measure for preventive service delivery to infants in primary care.	Brown CM, Samaan ZM, Morehous JF, Perkins AA, Kahn RS, Mansour ME.	J Eval Clin Pract.	2015	United States	H	Urban	Quantitative
Well-child care clinical practice redesign for serving low-income children.	Coker TR, Moreno C, Shekelle PG, Schuster MA, Chung PJ.	Pediatrics	2014	United States	H	Both	Mixed
Feasibility and Acceptability in a Community-Partnered Implementation of CenteringParenting for Group Well-Child Care.	Jones KA, Do S, Porras-Javier L, Contreras S, Chung PJ, Coker TR.	Acad Pediatr.	2018	Canada	H	Urban	Mixed

Continuity of Care in Infancy and Early Childhood Health Outcomes.	Enlow E, Passarella M, Lorch SA.	Pediatrics	2017	United States	H	Urban	Quantitative
Key role in the prevention of child neglect and abuse in Germany: continuous care by qualified family midwives.	Ayerle GM, Makowsky K, Schücking BA.	Midwifery	2012	Germany	H	Both	Mixed
Child health care utilisation in families with young or single mothers in a Swedish county.	Wallby T, Modin B, Hjern A.	J child Heal care Prof Work with Child Hosp community.	2013	Sweden	H	Both	Quantitative
A Program Model Describing a Community-Based Mother and Infant Health Program.	Guo Y, Rousseau J, Bender M, Lee J-A, Pimentel P, Bojorquez Y, et al.	Res Theory Nurs Pract.	2019	United States	H		Qualitative
Effects of nurse-led child health service in child-care centers: A survey study.	Kim SS, Roh YS.	Nurs Health Sci.	2018	South Korea	H	Urban	Quantitative
A Parent Coach Model for Well-Child Care Among Low-Income Children: A Randomized Controlled Trial.	Coker TR, Chacon S, Elliott MN, Bruno Y, Chavis T,	Pediatrics	2016	United States	H	Urban	Quantitative

	Biely C, et al.						
Implementation of a Preventive Services Bundle in Academic Pediatric Primary Care Centers.	Samaan ZM, Brown CM, Morehous J, Perkins AA, Kahn RS, Mansour ME.	Pediatrics.	2016	United States	H	Urban	Quantitative
Improving the immunization and health status of children in the Women, Infants, and Children (WIC) Program.	Shefer A, Smith PJ.	J Health Care Poor Underserved	2004	United States	H	Both	Quantitative
Integration of child health information systems: current state and local health department efforts.	Fehrenbach SN, Kelly JCR, Vu C.	J Public Health Manag Pract.	2004	United States	H	Both	Qualitative
A practice-based intervention to enhance quality of care in the first 3 years of life the - Healthy steps for young children program.	Minkovitz CS, Hughart N, Strobino D, Scharfstein D, Grason H, Hou W, et al.	JAMA-JOURNAL Am Med Assoc.	2003	United States	H	Both	Quantitative
**Upper middle income countries**							

Nascer no Brasil: continuity of care during pregnancy and postpartum period for women and newborns.	de Azevedo Bittencourt SD, Cunha EM, Soares Madeira Domingues RM, Soares Dias BA, Bastos Dias MA, Torres JA, et al.	Rev Saude Publica.	2020	Brazil	UM	Both	Mixed
Effect of basic public health service project on neonatal health services and neonatal mortality in China: a longitudinal time-series study.	Zhao P, Han X, You L, Zhao Y, Yang L, Liu Y.	BMJ Open.	2020	China	UM	Both	Quantitative
Fragmentation of maternal, child and HIV services: A missed opportunity to provide comprehensive care.	Haskins LJ, Phakathi SP, Grant M, Mntambo N, Wilford A, Horwood CM.	African J Prim Heal care Fam Med.	2016	South Africa	UM	Rural	Qualitative
Participatory development of a minimum dataset for the Khayelitsha district.	Mash B, Mahomed H.	S Afr Med J.	2000	South Africa	UM	Urban	Mixed

Cross-sectional study of a child health care programme at one family practice centre in Saudi Arabia.	Khattab MS.	East Mediterr Heal J	2000	Saudi Arabia	UM	Urban	Mixed
Impacts 2 years after a scalable early childhood development intervention to increase psychosocial stimulation in the home: A follow-up of a cluster randomised controlled trial in Colombia.	Andrew A, Attanasio O, Fitzsimons E, Grantham-McGregor S, Meghir C, Rubio-Codina M.	PLoS Med.	2018	Colombia	UM	Urban	Quantitative
Using the infrastructure of a conditional cash transfer program to deliver a scalable integrated early child development program in Colombia: cluster randomized controlled trial.	Attanasio OP, Fernández C, Fitzsimons EOA, Grantham-McGregor SM, Meghir C, Rubio-Codina M.	BMJ	2014	Colombia	UM	Urban	Quantitative
The crowded space of local accountability for maternal, newborn and child health: a case study of	Mukinda FK, Van Belle S, George A, Schneider H.	Health Policy Plan.	2020	South Africa	UM	Both	Mixed

the South African health system.							
Preventive healthcare of infants in a region of Lebanon: Parental beliefs, attitudes and behaviors.	Atiyeh GN, El-Mohandes A.	Matern Child Health J.	2005	Lebanon	UM	Both	Qualitative
Monitoring well-baby visits in primary healthcare facilities in a middle-income country.	Sokhela DG, Sibiya MN, Gwele NS.	SOUTH AFRICAN J CHILD Heal.	2018	South Africa	UM	Both	Quantitative
Forecasting the Value for Money of Mobile Maternal Health Information Messages on Improving Utilization of Maternal and Child Health Services in Gauteng, South Africa: Cost-Effectiveness Analysis.	LeFevre A, Cabrera-Escobar MA, Mohan D, Eriksen J, Rogers D, Parsons AN, et al.	JMIR MHEALTH UHEALTH.	2018	South Africa	UM	Urban	Quantitative
Integrated Information System for Early Detection of Maternal Risk Factors Based on Continuum of Care Approach of Mother and	DAMAYANTI N-A, SETIJANTO D, HARGONO A, WULANDARI R-D, SANTI M-	Healthc Inform Res	2019	Indonesia	UM	Both	Qualitative

Toddler Cohorts TT - 대한의료정보학회지. [Internet].	W, TJAHJONO B, et al.						
“You must leave but I didn’t want to leave”: qualitative evaluation of the integration of ART into postnatal maternal and child health services in Cape Town, South Africa.	Pellowski JA, Weber AZ, Phillips TK, Brittain K, Zerbe A, Abrams EJ, et al.	AIDS CARE-PSYCHOLOGICAL SOCIO-MEDICAL Asp AIDS/HIV.	2020	South Africa	UM	Urban	Mixed
Comparison of preventive health service provision before and after reorganization of primary care in Turkey: a community-based study.	Kaya ÇA, Akman M, Ünalan PC, Çifçili S, Uzuner A, Akdeniz E.	Prim Health Care Res Dev [Internet].	2019	Turkey	UM	Urban	Quantitative
Exploring the care provided to mothers and children by community health workers in South Africa: missed opportunities to provide comprehensive care.	Wilford A, Phakathi S, Haskins L, Jama NA, Mntambo N, Horwood C.	BMC Public Health.	2018	South Africa	UM	Rural	Qualitative

Assessing the care of children under one year old in Primary Health Care.	Furtado MC de C, Braz JC, Pina JC, de Mello DF, de Lima RAG.	Rev Lat Am Enfermage m.	201 3	Brazil	UM	Urba n	Mixed
Designing and Implementing an Early Childhood Health and Development Program in Rural, Southwest Guatemala: Lessons Learned and Future Directions.	Domek GJ, Cunningh am M, Jimenez-Zambran o A, Dunn D, Abdel-Maksoud M, Bronsert M, et al.	Adv Pediatr.	201 7	Guatemala	UM	Rura l	Mixed
Prevention of mother to child transmission of HIV (PMTCT) programme in KwaZulu-Natal, South Africa: an evaluation of PMTCT implementation and integration into routine maternal, child and women's health services.	Horwood C, Haskins L, Vermaak K, Phakathi S, Subbaye R, Doherty T.	Trop Med Int Health.	201 0	South Africa	UM	Both	Quantita tive
To evaluate if increased supervision and support of South African	Rotheram -Borus MJ, Le Roux K, Le Roux	Trials	201 7	South Africa	UM	Rura l	Quantita tive

Government health workers' home visits improves maternal and child outcomes: study protocol for a randomized control trial.	IM, Christodoulou J, Laurenzi C, Mbewu N, et al						
Missed opportunities in child healthcare.	Jonker L, Stellenberg EL.	African J Prim Heal care Fam Med.	2014	South Africa	UM	Urban	Qualitative
Coverage, quality of and barriers to postnatal care in rural Hebei, China: a mixed method study.	Chen L, Qiong W, van Velthoven MH, Yanfeng Z, Shuyi Z, Ye L, et al.	BMC Pregnancy Childbirth.	2014	China	UM	Rural	Mixed
Female clients' gender preferences for frontline health workers who provide maternal, newborn and child health (MNCH) services at primary health care level in Nigeria.	Okereke E, Unumeri G, Akinola A, Eluwa G, Adebajo S.	BMC Health Serv Res	2020	Nigeria	UM	Both	Quantitative
Challenges in access and satisfaction with reproductive, maternal, newborn and	Balogun M, Banke-Thomas A, Sekoni A,	PLoS One	2021	Nigeria	UM	Urban	Mixed

child health services in Nigeria during the COVID-19 pandemic: A cross-sectional survey.	Boateng GO, Yesufu V, Wright O, et al.						
The use of maternal and child health services in three population-based cohorts in Southern Brazil, 1982-2004.	Cesar JA, Matijasevich A, Santos IS, Barros AJD, Dias-da-Costa JS, Barros FC, et al.	Cad Saude Publica	2008	Brazil	UM	Urban	Quantitative
Child health services during a COVID-19 outbreak in KwaZulu-Natal Province, South Africa.	Jensen C, McKerrow NH.	South African Med J	2020	South Africa	UM	Both	Quantitative
A continuous quality improvement intervention to improve the effectiveness of community health workers providing care to mothers and children: a cluster randomised controlled trial in South Africa.	Horwood C, Butler L, Barker P, Phakathi S, Haskins L, Grant M, et al.	Hum Resour Health.	2020	South Africa	UM	Rural	Mixed
Quality of infant care in primary health services in Southern and	dos Santos AS, Duro SMS,	Rev Saude Publica.	2018	Brazil	UM	Both	Quantitative

Northeastern Brazil.	Cade N V, Facchini LA, Tomasi E.						
The experiences of early postpartum Shenzhen mothers and their need for home visit services: a qualitative exploratory study.	Xiao X, Ngai FW, Zhu SN, Loke AY.	BMC Pregnancy Childbirth.	2019	China	UM	Urban	Qualitative
**Low-middle income countries**							
Barriers to access and utilization of maternal and infant health services in Migori, Kenya.	Cheptum J, Gitonga M, Mutua E, Mukui S, Ndambuki J, Koima W.	Dev Ctry Stud	2014	Kenya	LM	Urban	Mixed
The coverage of continuum of care in maternal, newborn and child health: a cross-sectional study of woman-child pairs in Ghana. 2018 Jul;3(4).	Shibanuma A, Yeji F, Okawa S, Mahama E, Kikuchi K, Narh C, et al.	BMJ Glob Heal.	2018	Ghana	LM	Urban	Mixed
Persistent barriers to the use of maternal, newborn and child health services in Garissa sub-county,	Kisiangani I, Elmi M, Bakibinga P, Mohamed SF, Kisia	BMC Pregnancy Childbirth.	2020	Kenya	LM	Urban	Mixed

Kenya: a qualitative study.	L, Kibe PM, et al.						
Knowledge, utilization, and accessibility of child welfare card among caregivers in a tertiary center in South West Nigeria.	Alao MA, Olasinde YT, Agelebe E, Asinobi AO, Gbadero DA.	Niger J Clin Pract.	2020	Nigeria	LM	Urban	Quantitative
A retrospective review of the Honduras AIN-C program guided by a community health worker performance logic model.	Rodríguez DC, Peterson LA.	Hum Resour Health.	2016	Honduras	LM	Both	Mixed
Effect and feasibility of district level scale up of maternal, newborn and child health interventions in Pakistan: a quasi-experimental study.	Memon ZA, Muhammad S, Soofi S, Khan N, Akseer N, Habib A, et al.	BMJ Open.	2020	Pakistan	LM	Rural	Mixed
Measuring universal health coverage in reproductive, maternal, newborn and child health: An update of the composite coverage index.	Wehrmeister FC, Barros AJD, Hosseinpoor AR, Boerma T, Victora CG.	PLoS One.	2020	LMIC	LM	Both	Quantitative

Cultural beliefs and health-seeking practices: Rural Zambians' views on maternal-newborn care.	Buser JM, Moyer CA, Boyd CJ, Zulu D, Ngoma-Hazemba A, Mtenje JT, et al.	Midwifery	2020	Zambia	LM	Rural	Qualitative
Grandmothers as gatekeepers? The role of grandmothers in influencing health-seeking for mothers and newborns in rural northern Ghana.	Gupta ML, Aborigo RA, Adongo PB, Rominski S, Hodgson A, Engmann CM, et al	Glob Public Health	2015	Ghana	LM	Rural	Qualitative
Impact of a community-based integrated management of childhood illnesses (IMCI) programme in Gegharkunik, Armenia.	Thompson ME, Harutyunyan TL.	Health Policy Plan.	2009	Armenia	LM	Urban	Mixed
Knowledge of neonatal danger signs among mothers attending well baby clinic in Nakuru Central District, Kenya: cross sectional descriptive study.	Kibaru EG, Otara AM.	BMC Res Notes	2016	Kenya	LM	Urban	Quantitative
Quality of Care for Maternal and Newborn Health	Kc A, Singh DR, Upadhyay	Matern Child Health J.	2020	Nepal	LM	Both	Quantitative

in Health Facilities in Nepal.	a MK, Budhathoki SS, Gurung A, Målqvist M.						
The effectiveness of community-based interventions to improve maternal and infant health in the Northeast of Brazil.	Emond A, Pollock J, Da Costa N, Maranhão T, Macedo A.	Rev Panam Salud Publica.	2002	Brazil	LM	Urban	Quantitative
Integrating Nutrition Interventions into an Existing Maternal, Neonatal, and Child Health Program Increased Maternal Dietary Diversity, Micronutrient Intake, and Exclusive Breastfeeding Practices in Bangladesh: Results of a Cluster-Randomized Program Eva.	Nguyen PH, Kim SS, Sanghvi T, Mahmud Z, Tran LM, Shabnam S, et al.	J Nutr.	2017	Bangladesh	LM	Both	Mixed
Neonatal health program management in a resource-constrained setting in rural	Thomas A, Kumar V, Bhandari M, Ahuja RC, Singh	Int J Health Plann Manage.	2009	India	LM	Rural	Qualitative

Uttar Pradesh, India.	P, Baqui AH, et al.						
Predictors of Essential Health and Nutrition Service Delivery in Bihar, India: Results From Household and Frontline Worker Surveys.	Kosec K, Avula R, Holtemeyer B, Tyagi P, Hausladen S, Menon P.	Glob Heal Sci Pract.	2015	India	LM	Rural	Quantitative
Early results of an integrated maternal, newborn, and child health program, Northern Nigeria, 2009 to 2011.	Findley SE, Uwemedimo OT, Doctor H V, Green C, Adamu F, Afenyadu GY.	BMC Public Health.	2013	Nigeria	LM	Rural	Quantitative
Maternal, neonatal, and child health systems under rapid urbanization: a qualitative study in a suburban district in Vietnam.	Heo J, Kim SY, Yi J, Yu S-Y, Jung DE, Lee S, et al.	BMC Health Serv Res.	2020	Vietnam	LM	Urban	Qualitative
Factors affecting the implementation of health legislation and its impact on the rural poor in China: A case study of	Tolhurst R, Zhang TH, Yang H, Gao J, Tang SL.	Int J Health Plann Manage.	2004	China	LM	Rural	Mixed

implementation of the maternal and infant health care law in two poor counties.							
mHealth intervention “ImTeCHO” to improve delivery of maternal, neonatal, and child care services-A cluster-randomized trial in tribal areas of Gujarat, India.	Modi D, Dholakia N, Gopalan R, Venkatraman S, Dave K, Shah S, et al	PLOS Med.	2019	India	LM	Rural	Quantitative
An evaluation of skill and knowledge in delivery of reproductive and child health services by female health workers in Jamnagar District and corporation area, Gujarat state, India.	Makwana NR, Shah VR, Yadav S.	Int J Heal Allied Sci [Internet].	2012	India	LM	Both	Quantitative
The experience of men who participated in interventions to improve demand for and utilization of maternal and child health services in northern Nigeria: A qualitative	Oguntunde O, Nyenwa J, Yusuf FM, Dauda DS, Salihu A, Sinai I.	Reprod Health	2019	Nigeria	LM	Both	Qualitative

comparative study.							
Actions on social determinants and interventions in primary health to improve mother and child health and health equity in Morocco.	Boutayeb W, Lamlili M, Maamri A, Ben El Mostafa S, Boutayeb A.	Int J Equity Health.	2016	Morocco	LM	Both	Quantitative
The coverage of continuum of care in maternal, newborn and child health: A cross-sectional study of woman-child pairs in Ghana.	Shibanuma A, Yeji F, Okawa S, Mahama E, Kikuchi K, Narh C, et al.	BMJ Glob Heal [Internet]	2018	Ghana	LM	Urban	Quantitative
Improving maternal and newborn health: effectiveness of a community health worker program in rural Kenya.	Adam MB, Dillmann M, Chen M, Mbugua S, Ndung'u J, Mumbi P, et al.	PLoS One.	2014	Kenya	LM	Rural	Quantitative
The role of home-based records in the establishment of a continuum of care for mothers, newborns, and children in Indonesia.	Osaki K, Hattori T, Kosen S.	Glob Health Action	2013	Indonesia	LM	Both	Quantitative

India's Integrated Child Development Services programme; equity and extent of coverage in 2006 and 2016.	Chakrabarti S, Raghunathan K, Alderman H, Menon P, Nguyen P	Bull World Health Organ.	2019	India	LM	Both	Quantitative
An assessment of facilities and services at Anganwadi centers under the Integrated Child Development Service scheme in Northeast District of Delhi, India.	Malik A, Bhilwar M, Rustagi N, Taneja DK.	Int J Qual Heal care J Int Soc Qual Heal Care.	2015	India	LM	Urban	Quantitative
Assessing the operational effectiveness of a maternal and child health (MCH) conditional cash transfer pilot programme in Nigeria.	Oduenyi C, Ordu V, Okoli U.	BMC Pregnancy Childbirth.	2019	Nigeria	LM	Both	Quantitative
Accelerating progress for complementary feeding in Kenya: Key government actions and the way forward.	Ahoya B, Kavle JA, Straubinger S, Gathi CM.	Matern Child Nutr	2019	Kenya	LM	Both	Mixed
Incremental health system reform policy: Ecuador's law for the provision of free maternity	Chiriboga SR	J Ambul Care Manage.	2009	Ecuador	LM	Both	Quantitative

and child care 2009;32(2):80–90.							
Impact of the Maternal and Child Health handbook in Angola for improving continuum of care and other maternal and child health indicators: study protocol for a cluster randomised controlled trial.	Balogun OO, Tomo CK, Mochida K, Mikami M, da Rosa Vasconcelos H, Neves I, et al.	Trials	2020	Angola	LM	Both	Mixed
Using quality improvement methods to test and scale up a new national policy on early post-natal care in Ghana.	Twum-Danso NA, Dasoberi IN, Amenga-Etego IA, Adondiwo A, Kanyoke E, Boadu RO, et al	Health Policy Plan.	2014	Ghana	LM	Rural	Mixed
Perceived barriers to utilizing maternal and neonatal health services in contracted-out versus government-managed health facilities in the	Riaz A, Zaidi S, Khowaja AR.	Int J Heal policy Manag.	2015	Pakistan	LM	Rural	Qualitative

rural districts of Pakistan.							
Building on community outreach for childhood vaccination to deliver maternal and child health services in Laos: a feasibility assessment.	Jacobs B, Lindelow M, Xayyavong P, Sackett P.	Reprod Health Matters.	2012	Lao	LM	Rural	Qualitative
Female health workers at the doorstep: a pilot of community-based maternal, newborn, and child health service delivery in northern Nigeria.	Uzondu CA, Doctor H V, Findley SE, Afenyadu GY, Ager A.	Glob Heal Sci Pract.	2015	Nigeria	LM	Rural	Mixed
Postpartum family planning integration with maternal, newborn and child health services: a cross-sectional analysis of client flow patterns in India and Kenya.	Mackenzie D, Pfitzer A, Maly C, Waka C, Singh G, Sanyal A.	BMJ Open	2018	Kenya, India	LM,LM	Both	Quantitative
The effect of enhanced public-private partnerships on Maternal, Newborn and child Health Services and	Bakibinga P, Ettarh R, Ziraba AK, Kyobutungi C, Kamande	BMJ Open.	2014	Kenya	LM	Urban	Mixed

outcomes in Nairobi-Kenya: the PAMANECH quasi-experimental research protocol.	E, Ngomi N, et al.						
Advocacy for free maternal and child health care in Nigeria--Results and outcomes.	Okonofua F, Lambo E, Okeibunor J, Agholor K.	Health Policy.	2011	Nigeria	LM	Both	Mixed
Baby-Friendly Community Initiative-From national guidelines to implementation: A multisectoral platform for improving infant and young child feeding practices and integrated health services.	Kavle JA, Ahoya B, Kiige L, Mwando R, Olwenyi F, Straubinger S, et al.	Matern Child Nutr.	2019	Kenya	LM	Both	Mixed
Continuum of Care Services for Maternal and Child Health using mobile technology - a health system strengthening strategy in low and middle income countries.	Balakrishnan R, Gopichandran V, Chaturvedi S, Chatterjee R, Mahapatra T, Chaudhuri I.	BMC Med Inform Decis Mak.	2016	India	LM	Both	Quantitative

Community resource centres to improve the health of women and children in informal settlem ents in Mumbai: a cluster-randomised, controlled trial. .	More NS, Das S, Bapat U, Alcock G, Manjrekar S, Kamble V, et al.	Lancet Glob Heal	2017	India	LM	Urban	Quantitative
Overcoming barriers to access and utilization of maternal, newborn and child health services in northern Nigeria: an evaluation of facility health committees.	Oguntunde O, Surajo IM, Dauda DS, Salihu A, Anas-Kolo S, Sinai I.	BMC Health Serv Res.	2018	Nigeria	LM	Both	Qualitative
Enhancing the Knowledge and Behaviors of Fieldworkers to Promote Family Planning and Maternal, Newborn, and Child Health in Bangladesh Through a Digital Health Training Package: Results From a Pilot Study.	J Limaye R, Ballard Sara A, Ahmed N, Ohkbuo S, Deka S, Mickish Gross C, et al.	Int Q Community Health Educ.	2020	Bangladesh	LM	Both	Quantitative
**Low-income countries**							
Acceptability and trust of community health workers	Singh D, Cumming R, Negin J.	Health Educ Res	2015	Uganda	L	Rural	Qualitative

offering maternal and newborn health education in rural Uganda.							
A job analysis of community health workers in the context of integrated nutrition and early child development.	Phuka J, Maleta K, Thomas M, Gladstone M.	Ann N Y Acad Sci.	2014	Malawi	L	Both	Qualitative
Benefit or burden: introducing paraprofessional support staff to health visiting teams: the case of Starting Well.	Mackenzie M.	Health Soc Care Community.	2006	Sierra Leone	L	Urban	Mixed
Engaging community health workers in maternal and newborn care in eastern Uganda.	Okuga M, Kemigisa M, Namutamba S, Namazzi G, Waiswa P.	Glob Health Action.	2015	Uganda	L	Both	Qualitative
Contribution of traditional birth attendants to the formal health system in Ethiopia: the case of Afar region.	Temesgen TM, Umer JY, Buda DS, Haregu TN.	Pan Afr Med J.	2012	Ethiopia	L	Both	Qualitative

Community participation to improve health services for children: a methodology for a community dialogue intervention in Uganda.	Muhwezi WW, Palchik EA, Kiwanuka DH, Mpanga F, Mukundane M, Nanungi A, et al.	Afr Health Sci.	2019	Uganda	L	Rural	Qualitative
Supportive supervision and constructive relationships with healthcare workers support CHW performance: Use of a qualitative framework to evaluate CHW programming in Uganda.	Ludwick T, Turyakira E, Kyomuhangi T, Manalili K, Robinson S, Brenner JL.	Hum Resour Health.	2018	Uganda	L	Rural	Mixed
Cohort profile : Bandim Health Project's (BHP) rural Health and Demographic Surveillance System (HDSS)-a nationally representative HDSS in Guinea-Bissau.	Thysen SM, Fernandes M, Benn CS, Aaby P, Fisker AB.	BMJ Open	2019	Guinea-Bissau	L	Rural	Mixed
Community based maternal and child health care in Nepal: self-reported	Chhetry S, Clapham S, Basnett I	JNMA J Nepal Med Assoc.	2005	Nepal	L	Both	Quantitative

performance of Maternal and Child Health Workers.							
Impact of the integrated package of nutrition and health services	Saiyed F, Seshadri S.	Indian J Pediatr.	200 0	India	L	Urba n	Mixed
Home-based neonatal care: summary and applications of the field trial in rural Gadchiroli, India (1993 to 2003).	Bang AT, Bang RA, Reddy HM.	J Perinatol.	200 5	India	L	Rura l	Quantita tive
Integration of maternal postpartum services in maternal and child health services in Kaya health district (Burkina Faso): an intervention time trend analysis.	Belemsag a DY, Goujon A, Tougri H, Coulibaly A, Degomm e O, Duysburg h E, et al.	BMC Health Serv Res.	201 8	Burkina Faso	L	Both	Mixed
Immediate and sustained effects of user fee exemption on healthcare utilization among children under five in Burkina Faso: A controlled interrupted time-series analysis.	Zombre D, De Allegri M, Ridde V.	Soc Sci Med.	201 7	Burkina Faso	L	Rura l	Quantita tive
Integration of postpartum care into child health and immunization	Belemsag a DY, Goujon A, Bado	Reprod Health	201 8	Burkina Faso	L	Both	Mixed

services in Burkina Faso: findings from a cross-sectional study.	A, Kouanda S, Duysburgh E, Temmerman M, et al.						
A cluster randomised controlled trial of the community effectiveness of two interventions in rural Malawi to improve health care and to reduce maternal, newborn and infant mortality.	Lewycka S, Mwansambo C, Kazembe P, Phiri T, Mganga A, Rosato M, et al.	Trials	2010	Malawi	L	Rural	Mixed
Integrating reproductive and child health services enables access to modern contraception in Sierra Leone.	Koroma AS, Ghatahora SK, Ellie M, Kargbo A, Jalloh UH, Kandeh A, et al.	Int J Health Plann Manage	2019	Sierra Leone	L	Both	Mixed
Protocol for the evaluation of a complex intervention aiming at increased utilisation of primary child health services in Ethiopia: a before and after study in intervention and	Berhanu D, Okwaraji YB, Belayneh AB, Lemango ET, Agonafer N, Birhanu BG, et al.	BMC Health Serv Res	2020	Ethiopia	L	Both	Quantitative

comparison areas.							
Better than nothing? maternal, newborn, and child health services and perinatal mortality, Lubumbashi, democratic republic of the Congo: a cohort study.	Ntambue AM, Malonga FK, Dramaix-Wilmet M, Ngatu RN, Donnen P.	BMC Pregnancy Childbirth.	2016	Congo	L	Urban	Quantitative
Expanded Quality Management Using Information Power (EQUIP): protocol for a quasi-experimental study to improve maternal and newborn health in Tanzania and Uganda	Hanson C, Waiswa P, Marchant T, Marx M, Manzi F, Mbaruku G, et al.	. Implement Sci.	2014	Tanzania, Uganda	L,L	Both	Mixed
Process evaluation of the community-based newborn care program implementation in Geze Gofa district, south Ethiopia: a case study evaluation design.	Gebremedhin T, Daka DW, Alemayehu YK, Yitbarek K, Debie A.	BMC Pregnancy Childbirth.	2019	Ethiopia	L	Both	Mixed
Who is to blame? Perspectives of	Opwora AS, Laving	BMC Public Health.	2011	Kenya	L	Rural	Qualitative

caregivers on barriers to accessing healthcare for the under-fives in Butere District, Western Kenya.	AMR, Nyabola LO, Olenja JM.						
The role of the traditional leader in implementing maternal, newborn and child health policy in Malawi.	Walsh A, Matthew s A, Manda-Taylor L, Brugha R, Mwale D, Phiri T, et al.	Health Policy Plan.	201 8	Malawi	L	Rura l	Both
Enhancing the routine health information system in rural southern Tanzania: success es, challenges and lessons learned.	Maokola W, Willey BA, Shirima K, Chemba M, Armstron g Schellenb erg JRM, Mshinda H, et al.	Trop Med Int Health.	201 1	Tanzania	L	Rura l	Quantita tive
Integrating HIV and Maternal, Neonatal and Child Health Services in Rural Malawi: An Evaluation of the Implementation Processes and Challenges.	Gunda A, Jousset A, Tchereni T, Joseph J, Mwapasa V.	J Acquir Immune Defic Syndr.	201 7	Malawi	L	Rura l	Quantita tive

Is the role of Health Extension Workers in the delivery of maternal and child health care services a significant attribute? The case of Dale district, southern Ethiopia.	Negussie A, Girma G.	BMC Health Serv Res.	2012	Ethiopia	L	Rural	Mixed
The human resource implications of improving financial risk protection for mothers and newborns in Zimbabwe.	Chirwa Y, Witter S, Munjoma M, Mashange W, Ensor T, McPake B, et al.	BMC Health Serv Res.	2013	Zimbabwe	L	Both	Mixed
Effect on maternal and child health services in Rwanda of payment to primary health-care providers for performance: an impact evaluation.¨	Basinga P, Gertler PJ, Binagwaho A, Soucat ALB, Sturdy J, Vermeersch CMJ.	Lancet	2011	Rwanda	L	Both	Quantiative
Effectiveness of Kenya's Community Health Strategy in delivering community-based maternal and newborn health care in	Wangalwa G, Cudjoe B, Wamalwa D, Machira Y, Ofware P,	Pan Afr Med J.	2012	Kenya	L	Both	Quantitative

Busia County, Kenya: non-randomized pre-test post test study.	Ndirangu M, et al.						
Community-based intervention packages facilitated by NGOs demonstrate plausible evidenc e for child mortality impact.	Ricca J, Kureshy N, LeBan K, Prosnitz D, Ryan L.	Health Policy Plan.	201 4	Benin, Cambodia, Ethiopia, Guinea, Haiti, Malawi, Mali, Nepal, Rwanda	L, L, L, L, L, L, L, L, L	Both	Quantita tive
Can volunteer community health workers manage multiple roles? An interrupted time-series analysis of combined HIV and maternal and child health promotion in Iringa, Tanzania.	Shelley KD, Frumence G, Mpembe ni R, George AS, Stuart EA, Killewo J, et al	Health Policy Plan.	201 8	Tanzania	L	Rura l	Quantita tive
Assessing the Integrated Community-Based Health Systems Strengthening initiative in northern Togo: a pragmatic effectiveness-implementation study protocol.	Lauria ME, Fiori KP, Jones HE, Gbeleou S, Kenkou K, Agoro S, et al.	Implement Sci.	201 9	Togo	L	Both	Mixed

Profile, knowledge, and work patterns of a cadre of maternal, newborn, and child health CHWs focusing on preventive and promotive services in Morogoro Region, Tanzania.	LeFevre AE, Mpembeni R, Chitama D, George AS, Mohan D, Urassa DP, et al.	Hum Resour Health.	2015	Tanzania	L	Both	Quantitative
**Additional**							
LiST as a catalyst in program planning: experiences from Burkina Faso, Ghana and Malawi.	Bryce J, Friberg IK, Kraushaar D, Nsona H, Afenyadu GY, Nare N, et al.	Int J Epidemiol.	2010	Burkina Faso, Ghana, Malawi	L,LM,L	Both	Quantitative
Tackling the hard problems: implementation experience and lessons learned in newborn health from the African Health Initiative.	Magge H, Chilengi R, Jackson EF, Wagenaar BH, Kante AM.	BMC Health Serv Res.	2017	Countries: Ghana, Mozambique, Rwanda, Tanzania, and Zambia	LM, L, L, L, LM	Both	Mixed
The contribution of child health days to improving coverage of periodic interventions in six African countries.	Oliphant NP, Mason JB, Doherty T, Chopra M, Mann P, Tomlinson M, et al.	Food Nutr Bull	2010	Ethiopia, Madagascar, Tanzania, Uganda, Zambia, and Zimbabwe	L, L, L,L, LM, L	Rural	Mixed

Improving team-based care for children: shared well child care involving family practice nurses.	Warmels G, Johnston S, Turley J	Prim Health Care Res Dev	2017				Mixed
Research priorities to reduce global mortality from newborn infections by 2015.	Bahl R, Martines J, Ali N, Bhan MK, Carlo W, Chan KY, et al.	Pediatr Infect Dis J.	2015	Global		Both	Quantitative
Prioritizing research for integrated implementation of early childhood development and maternal, newborn, child and adolescent health and nutrition platforms.	Sharma R, Gaffey MF, Alderman H, Bassani DG, Bogard K, Darmstadt GL, et al.	J Glob Health.	2017	Global			Quantitative
Integrating a Parenting Intervention With Routine Primary Health Care: A Cluster Randomized Trial.	Chang SM, Grantham-McGregor SM, Powell CA, Vera-Hernández M, Lopez-Boo F, Baker-Henningh	Pediatrics.	2015	Jamaica, Antigua, St Lucia	UM, H, UM	Both	Quantitative

	am H, et al.						
Opportunities to improve postpartum care for mothers and infants: design of context-specific packages of postpartum interventions in rural districts in four sub-Saharan African countries.	Duysburgh E, Kerstens B, Kouanda S, Kaboré CP, Belemsaga Yugbare D, Gichangi P, et al.	BMC Pregnancy Childbirth.	2015	Burkina Faso, Kenya, Malawi, Mozambique	L, LM, L, L	Rural	Mixed
**Reviews**							
**Title**	**Authors**	**Journal/Publisher**	**Year**	**Country/Countries**	**World Bank Income Level**		
**High-income countries**							
Rethinking well-child care.	Schor EL.	Pediatrics	2004	United States	H		
CDC Kerala 1: Organization of Clinical Child Development Services	Nair MKC, George B, Harikumaran Nair GS, Bhaskaran D, Leena ML, Russell PSS.	Indian J Pediatr.	2014	India	H		
The Aboriginal Maternal and Infant Health Service: a decade	Murphy E, Best E.	N S W Public Health Bull.	2012	Australia	H		

of achievement in the health of women and babies in NSW							
Text4baby in the United States and Russia: an opportunity for understanding how mHealth affects maternal and child health.	Parker RM, Dmitrieva E, Frolov S, Gazmararian JA.	J Health Commun.	2012	Russia, United States	H		
Maternal and child health in Israel: building lives.	Rubin L, Belmaker I, Somekh E, Urkin J, Rudolf M, Honovich M, et al.	Lancet	2017	Israel	H		
Update on well-baby and well-child care from 0 to 5 years: What's new in the Rourke Baby Record?	Rourke L, Leduc D, Constantin E, Carsley S, Rourke J.	Can Fam Physician.	2010	Canada	H		
Rourke Baby Record 2017: Clinical update for preventive care of children up to 5 years of age.	Li P, Rourke L, Leduc D, Arulthas S, Rezk K, Rourke J.	Can Fam Physician.	2019	Canada	H		
Child development programme in Singapore 1988 to 2007.	Ho L-Y.	Ann Acad Med Singapore	2007	Singapore	H		

Theoretical, developmental & cultural orientations of school-based prevention progr ams for preschoolers.	Humphrie s ML, Keenan KE.	Clin Child Fam Psychol Rev.	200 6	United States	H		
Interim schedule for pregnant women and children during the COVID-19 pandemic.	Bogler T, Bogler O.	Can Fam Physician.	202 0	Canada	H		
Child health care centres: an academic model for comprehensive child health care in the community. 2005	Katz M, Urkin J, Bar-David Y, Cohen AH, Warshaw sky S, Barak N.	Child Care Health Dev.	200 5	Israel	H		
A review of the public-funded primary health care facilities for children in the pluralistic health care settings of Barbados, a Caribbean island.	Nielsen AL, Kumar A.	Prim Health Care Res Dev.	201 6	Barbados	H		
Rethinking well-child care in the United States: an international comparison. Pediatrics.	Kuo AA, Inkelas M, Lotstein DS, Samson KM,	Pediatrics	200 6	Australia, Canada, Denmark, England, France, Germany, Japan, Netherlands,	H		

	Schor EL, Halfon N.			Spain, Sweden			
Maternal and child health in the occupied Palestinian territory.	Rahim HFA, Wick L, Halileh S, Hassan-Bitar S, Chekir H, Watt G, et al.	Lancet	2009	Palestine			
Early childhood intervention programs in the US: recent advances and future recommendations.	Gray R, McCormick MC.	J Prim Prev	2005	United States	H		
Unmet Health Needs in Early Childhood in South Korea.	Shin H, Shim K, Hwang W.	J Community Health Nurs.	2020	South Korea	H		
Healthy starts for all: policy prescriptions.	Miller WD, Sadegh-Nobari T, Lillie-Blanton M.	Am J Prev Med.	2011	United States	H		
Preventive health services for young children in Israel: historical development and current challenges.	Zimmerman DR, Verbov G, Edelstein N, Stein-Zamir C.	Isr J Health Policy Res.	2019	Israel			
Early Childhood Home Visiting.	Duffee JH,	Pediatrics	2017	United States	H		

	Mendelsohn AL, Kuo AA, Legano LA, Earls MF, Pediat CC, et al.						
Well-child care: Effectiveness of current recommendations.	Dinkevich E, Ozuah PO.	Clin Pediatr	2002	United States	H		
A comparison of Child Health Programmes recommended for preschool children in selected high-income countries.	Wood R, Blair M.	Child Care Health Dev	2014		H		
A comparative analysis of early child health and development services and outcomes in countries with different redistributive policies.	van den Heuvel M, Hopkins J, Biscaro A, Srikanthan C, Feller A, Bremberg S, et al.	BMC Public Health.	2013	Cuba, Sweden, Canada, Netherlands, United States	UM, H, H, H, H		
Creating New Strategies to Enhance Postpartum Health and Wellness.	Cornell A, McCoy C, Stampfel C, Bonzon E, Verbiest S.	Matern Child Health J.	2016	United States	H		

Maternal and child health in Seychelles.	Govinden P, Henderson J, Rizvi Z, Seth V, Shamlaye H.	Neurotoxicology.	2020	Seychelles	H		
Measuring the Impact and Outcomes of Maternal Child Health Federal Programs.	Taylor YJ, Nies MA.	Matern Child Health J.	2013	United States	H		
Tensions in maternal and child health policy in Victoria: looking back, looking forward.	Keleher H, Reiger K.	Aust Health Rev	2004	Australia	H		
Aboriginal and Torres Strait Islander family access to continuity of health care services in the first 1000 days of life: a systematic review of the literature.	Sivertsen N, Anikeeva O, Deverix J, Grant J.	BMC Health Serv Res.	2020	Australia	H		
Pioneers, paediatricians and public health: The evolution of community child health services, Clifton, Nottingham 1983-1999.	Didcock E, Polnay L.	Public Health	2001	United Kingdom	H		
**Upper-middle income countries**							

Saving the lives of South Africa's mothers, babies, and children: can the health system deliver?	Chopra M, Daviaud E, Pattinson R, Fonn S, Lawn JE	Lancet	2009	South Africa	UM		
Child health in Cuba.	Rodriguez F V, Lopez NB, Choonara I.	Arch Dis Child.	2008	Cuba	UM		
Analysis of the maternal and child health care status in Suizhou City, Hubei Province, China, from 2005 to 2011.	Li C-L, Jiang T, Hu X-Z, Zhao K, Yu Q, Zhang H-P.	PLoS One.	2013	China	UM		
The Child Health Care System of Serbia.	Bogdanović R, Lozanović D, Pejović Milovančević M, Sokal Jovanović L.	J Pediatr.	2016	Serbia	UM		
The Child Health Care System of Macedonia.	Tasic V, Danilovski D, Gucev Z.	J Pediatr.	2016	Macedonia	UM		
Well child care: a comprehensive strategy for Cuban children and adolescents.	Esquivel M, Álvarez G, Izquierdo ME,	MEDICC Rev.	2014	Cuba	UM		

	Martínez D, Tamayo V.						
Reproductive, maternal, neonatal and child health in the 30 years since the creation of the Unified Health System (SUS).	Leal M do C, Szwarcwald CL, Almeida PVB, Aquino EML, Barreto ML, Barros F, et al.	Cien Saude Colet.	2018	Brazil	UM		
Comprehensive Care for Cuban Children in the First 1000 Days of Life.	Esquivel-Lauzurique M, Álvarez-Valdés G, Castro-Pacheco BL, Santana-Espinosa MC, Machado-Lubián MDC, Herrera-Alcázar V, et al.	MEDICC Rev.	2019	Cuba	UM		
Impact of health reforms on child health services in Europe: The case of Bulgaria.	Rechel B, Spencer N, Blackburn C, Holland R, Rechel B.	Eur J Public Health	2009	Bulgaria	UM		

Child Health Care in Georgia.	Margvelashvili L, Karseladze R, Abesadze G, Kvlividze O.	J Pediatr	2016	Georgia	UM		
The Medical Insurance for a New Generation: a viable answer for the health needs of Mexican children.	Muñoz-Hernández O, Chertorivski-Woldenberg S, Cortés-Gallo G, Pérez-Cuevas R	alud Publica Mex.	2012	Mexico	UM		
Reducing Child Mortality: The Contribution of Ceara State, Northeast of Brazil, on Achieving the Millennium Development Goal 4 in Brazil.	e Silva A, Correia LL, Campos JS, de Oliveira Andrade FM, da Silveira DM, Madeiro Leite AJ, et al.	Matern Child Health J	2015	Brazil	UM		
**Lower-middle income countries**							
Achieving Millennium Development Goals 4 and 5 in Sri Lanka.	Senanayake H, Goonewardene M, Ranatunga A,	BJOG	2011	Sri Lanka	LM		

	Hattotuwa R, Amarasekera S, Amarasinghe I.						
Contextual design choices and partnerships for scaling early child development pro grammes.	Milner KM, Bernal Salazar R, Bhopal S, Brentani A, Britto PR, Dua T, et al.	Arch Dis Child.	2019		LM		
Marching toward the Millennium Development Goals: what about health systems, health-seeking behaviours and health service utilization in Pakistan?	Shaikh BT.	World Health Popul.	2008	Pakistan	LM		
China: policy and practice of MCH since the early 1990s.	Guo Y, Zakus D, Liang H.	Matern Child Health J.	2008	China	LM		
Cross-National Systematic Review of Neonatal Mortality and Postnatal Newborn Care: Special Focus on Pakistan.	Ahmed M, Won Y.	Int J Environ Res Public Health	2017	Pakistan	LM		

CDC Kerala--The Untold Story.	Nair MKC, Leela LM, George B, Bhaskaran D, Pillai AN, Sarasamma HNGN.	Indian J Pediatr.	2016	India	LM		
**Low-income countries**							
Maternal and newborn health in Tanzania.	Winani K.	Int J Gynaecol Obstet Off organ Int Fed Gynaecol Obstet.	2011	Tanzania	L		
Achieving Millennium Development Goals 4 and 5 in Bangladesh.	Chowdhury S, Banu LA, Chowdhury TA, Rubayet S, Khatoon S.	BJOG	2011	Bangladesh	L		
Pakistan's maternal and child health policy: analysis, lessons and the way forward.	Siddiqi S, Haq IU, Ghaffar A, Akhtar T, Mahaini R.	Health Policy	2004	Pakistan	L		
Achieving Millennium Development Goals 4 and 5 in Nepal.	Malla DS, Giri K, Karki C, Chaudhary P	BJOG.	2011	Nepal	L		

Impact of a critical health workforce shortage on child health in Zimbabwe: a country case study on progress in child survival, 2000-2013.	Haley CA, Vermund SH, Moyo P, Kipp AM, Madzima B, Kanyowa T, et al.	Health Policy Plan.	2017	Zimbabwe	L		
Scaling up primary health services for improving reproductive, maternal, and child health: a multisectoral collaboration in the conflict setting of Afghanistan.	Das JK, Akseer N, Mirzazada S, Peera Z, Noorzada O, Armstrong CE, et al.	BMJ	2018	Afghanistan	L		
Somalia: supporting the child survival agenda when routine health service is broken.	Mirza IR, Kamadjeu R, Assegid K, Mulugeta A.	J Infect Dis.	2012	Somalia	L		
Reducing child mortality in India in the new millennium.	Claeson M, Bos ER, Mawji T, Pathmanathan I.	Bull World Health Organ.	2000	India	L		
**Regional - global**							
Programs for parents of infants and toddlers: recent evidence	Olds DL, Sadler L, Kitzman H.	J Child Psychol Psychiatry.	2007				

from randomized trials .							
Neonatal survival: a call for action.	Martines J, Paul VK, Bhutta ZA, Koblinsky M, Soucat A, Walker N, et al.	Lancet	2005	Global			
Quality improvement in maternal, neonatal and child health services in sub-Saharan Africa: A look at five resource-poor countries.	Mekbib T, Leatherman S.	Ethiop J Heal Dev.	2020	Countries: Ethiopia, Ghana, Nigeria, Tanzania, Uganda	L, LM, LM, LM, L		
Health services for children in western Europe.	Wolfe I, Thompson M, Gill P, Tamburlini G, Blair M, van den Bruel A, et al.	Lancet	2013				
Achieving child survival goals: potential contribution of community health workers.	Haines A, Sanders D, Lehmann U, Rowe AK, Lawn JE, Jan S, et al.	Lancet	2007	Global			

Special delivery: an analysis of mHealth in maternal and newborn health programs and their outcomes around the world.	Tamrat T, Kachnowski S.	Matern Child Health J.	2012	Global			
Midwifery-led antenatal care models: mapping a systematic review to an evidence-based quality framework to identify key components and characteristics of care.	Symon A, Pringle J, Cheyne H, Downe S, Hundley V, Lee E, et al.	BMC Pregnancy Childbirth.	2016	Global			
Linking families with pre-school children from healthcare services to community resources: a systematic review protocol.	Burns J, Conway DI, Gnich W, Macpherson LMD.	Syst Rev	2017	Global			
Countdown to 2015 for maternal, newborn, and child survival: the 2008 report on tracking coverage of interventions.	Bryce J, Daelmans B, Dwivedi A, Fauveau V, Lawn JE, Mason E, et al.	Lancet	2008	Global			
Community participation:	Rosato M,	Lancet	2008	Global			

lessons for maternal, newborn, and child health.	Laverack G, Grabman LH, Tripathy P, Nair N, Mwansambo C, et al.						
Alma-Ata 30 years on: revolutionary, relevant, and time to revitalise.	Lawn JE, Rohde J, Rifkin S, Were M, Paul VK, Chopra M.	Lancet	2008	Global			
Outcomes research in pediatric settings: Recent trends and future directions.	Forrest CB, Shipman SA, Dougherty D, Miller MR.	Pediatrics.	2003	Global			
Integration of immunization services with other health interventions in the developing world: what works and why? Systematic literature review.	Wallace A, Dietz V, Cairns KL	Trop Med Int Heal.	2009	Global			
Growth monitoring and promotion: review of evidence of impact.	Ashworth A, Shrimpton R, Jamil K.	Matern CHILD Nutr.	2008	Global			

Continuum of care for maternal, newborn, and child health: from slogan to service delivery.	Kerber KJ, Graft-Johnson JE de, Bhutta ZA, Okong P, Starrs A, Lawn JE.	Lancet	2007	Global			
Strategies for integrating primary health services in low- and middle-income countries at the point of delivery.	Dudley L, Garner P.	Cochrane Database Syst Re	2011	Global			
Primary care services promoting optimal child development from birth to age 3 years - Review of the literature.	Regalado M, Halfon N.	Arch Pediatr Adolesc Med.	2001	Global			
The evolution of child health programmes in developing countries: from targeting diseases to targeting people.	Claeson M, Waldman RJ.	Bull World Health Organ.	2000	Global			
The nature and impact of collaboration and integrated service delivery for pregnant women, children and families.	Schmied V, Mills A, Kruske S, Kemp L, Fowler C, Homer C.	J Clin Nurs	2010	Global			

Advancing newborn health: The Saving Newborn Lives initiative.	Tinker A, Parker R, Lord D, Grear K.	Glob Public Health.	2010	Global			
Measuring coverage in MNCH: indicators for global tracking of newborn care.	Moran AC, Kerber K, Sitrin D, Guenther T, Morrissey CS, Newby H, et al.	PLoS Med.	2013	Global			
Postpartum program actions in primary health care: an integrative review.	van den Heuvel M, Hopkins J, Biscaro A, Srikanthan C, Feller A, Bremberg S, et al.	Cien Saude Colet.	2019	Global			
Enhancing the child survival agenda to promote, protect, and support early child developme nt.	Jensen SKG, Bouhouch RR, Walson JL, Daelmans B, Bahl R, Darmstadt GL, et al.	Semin Perinatol.	2015	Global			
The Western Pacific Regional Child Survival	Jayawardena N,	J Paediatr Child Health.	2012				

Strategy: progress and challenges in implementation.	Subhi R, Duke T.						
Overview, methods and results of multi-country community-based maternal and newborn care economic analysis.	Daviaud E, Owen H, Pitt C, Kerber K, Bianchi Jassir F, Barger D, et al.	Health Policy Plan.	2017	Ethiopia, Ghana, South Africa, Tanzania, Uganda	L, LM, UM, L, L		
Impact of packaged interventions on neonatal health: a review of the evidence.	Haws RA, Thomas AL, Bhutta ZA, Darmstadt GL	Health Policy Plan.	2007	Global			
Challenges and opportunities: the health of women and newborns in the Russian Federation.	Dymchenko LD, Callister LC.	J Perinat Neonatal Nurs.	2002				
A description of birth in Finland.	Callister LC, Lauri S, Vehvilainen-Julkunen K.	MCN Am J Matern Child Nurs.	2000				
The implications of US experiences with early childhood interventions for the UK Sure Start Programme.	Gray R, Francis E.	Child Care Health Dev.	2007				

Evolution of a child health profile initiative.	Linzer DS, Lloyd-Puryear MA, Mann M, Kogan MD.	J Public Health Manag Pract.	2004				
Eliminating health inequities: national goals and developing programs.	Henry JK.	J Obstet Gynecol neonatal Nurs JOGN N.	2001				
Impact of Head Start's Entry Age and Enrollment Duration on Children's Health.	Lee K.	Soc work	2016				
Integration of HIV/AIDS services with maternal, neonatal and child health, nutrition, and family planning services.	Lindegren M Lou, Kennedy CE, Bain-Brickley D, Azman H, Creanga AA, Butler LM, et al.	Cochrane database Syst Rev.	2012				
Integrating early child development programs into health and nutrition services in Bangladesh: benefits and challenges	Hamadani JD, Nahar B, Huda SN, Tofail F.	Ann N Y Acad Sci.	2014				
Advancing newborn health	Marsh DR,	Marsh DR, Darmstadt	2002				

and survival in developing countries: a conceptual framework.	Darmstadt GL, Moore J, Daly P, Oot D, Tinker A.	GL, Moore J, Daly P, Oot D, Tinker A.					
Continuity of primary care clinician in early childhood. Pediatrics.	Inkelas M, Schuster MA, Olson LM, Park CH, Halfon N.	Pediatrics	2004				
Women's, children's, and adolescents' health in humanitarian and other crises.	Zeid S, Gilmore K, Khosla R, Papowitz H, Engel D, Dakkak H, et al.	BMJ	2015				
Economic and social factors are some of the most common barriers preventing women from accessing maternal and newborn child health (MNCH) and prevention of mother-to-child transmission (PMTCT) services: a literature review.	hIarlaithe MO, Grede N, de Pee S, Bloem M.	AIDS Behav.	2014	Global			
Reducing newborn mortality in the	Milner KM, Duke	J Paediatr Child Health.	2013				

Asia-Pacific region: Quality hospital services and community-based care.	T, Bucens I.						
Prevention of mother-to-child HIV transmission within the continuum of maternal, newborn, and child health services.	Chi BH, Bolton-Moore C, Holmes CB.	Curr Opin HIV AIDS.	2013	Global			
Every Newborn: health-systems bottlenecks and strategies to accelerate scale-up in countries.	Dickson KE, Simen-Kapeu A, Kinney M V, Huicho L, Vesel L, Lackritz E, et al.	Lancet	2014	Global			
A Review of e-Health Interventions for Maternal and Child Health in Sub-Sahara Africa.	Obasola OI, Mabawonku I, Lagunju I.	Matern Child Health J	2015				
Experiences integrating delivery of maternal and child health services with childhood immunization programs: systematic review update.	Wallace AS, Ryman TK, Dietz V.	J Infect Dis.	2012	Global			

Essential interventions: implementation strategies and proposed packages of care.	Lassi ZS, Kumar R, Mansoor T, Salam RA, Das JK, Bhutta ZA.	Reprod Health.	201 4	Global			
Effects of integrated child development and nutrition interventions on child developme nt and nutritional status.	Grantha m-McGregor SM, Fernald LCH, Kagawa RMC, Walker S.	Ann N Y Acad Sci.	201 4	Global			
